# Complete chloroplast genome sequence and phylogenetic analysis of *Symphytum officinale*

**DOI:** 10.1590/1678-4685-GMB-2024-0258

**Published:** 2025-06-30

**Authors:** Yuemei Zhao, Xiaodan Chen, Tao Zhou, Rongxiang Zhang, Fang Zhao, Xiao Zhang

**Affiliations:** 1 Guizhou Education University, School of Biological Sciences, Guiyang, Guizhou, China.; 2 Shanxi Normal University, College of Life Science, Taiyuan, Shanxi, China.; 3 Xi’an Jiaotong University, School of Pharmacy, Xi’an, Shaanxi, China.; 4 Institute of Botany of Shaanxi Province, Xi’an Botanical Garden of Shaanxi Province, Xi’an, Shaanxi, China.

**Keywords:** Symphytum officinale, chloroplast genome, comparative analysis, phylogenetic analyses

## Abstract

*Symphytum officinale* is a perennial herb belonging to the Boraginaceae. Here, we sequenced the complete chloroplast (cp) genomes of *S. officinale* using Illumina sequencing technology. The results revealed that the cp genome is 148,149 bp in length and exhibits a typical quadripartite structure, with a pair of inverted repeat regions (IR) comprising 27,001 bp, a large single-copy (LSC) region comprising 77,366 bp, and a small single-copy (SSC) region comprising 16,781 bp. The sequence contained 133 unique genes, including 86 protein-coding genes, 37 transfer RNAs, eight ribosomal RNAs, and two pseudogenes. Six tandem, 42 dispersed, and 38 simple sequence repeats were identified. Sequence divergence analysis across 21 Boraginaceae species revealed that the most divergent regions, potentially serving as specific DNA barcodes, were found in non-coding spacers. A comparative analysis of the IR/SC boundary regions of the 21 Boraginaceae species revealed IR expansion events in *S. officinale*. Phylogenetic analysis based on 63 protein-coding genes demonstrated that *S. officinale* was closely related to *Nonea vesicaria*. This represents the first cp genome sequenced in *Symphytum*, and our results provide valuable genetic information for future population and phylogenetic studies of Boraginaceae.

## Introduction

The chloroplast (cp) is a uniparentally inherited plant organelle which is comprised of approximately 130 genes and have a significant part in photosynthesis (Inoue, 2011). Generally, the cp genome is a circular DNA molecule characterized by the presence of a pair of inverted repeat sequences, which are flanked by a large single-copy region and a small single-copy region (Yang *et al*., 2010). In most cases, the gene structure and content of the cp genome are highly conserved, with sizes ranging from 120 to 160 kb. The chloroplast genome exhibits a moderate pace of nucleotide substitution (Zhang *et al*., 2017), and as a valuable dataset, the cp genome is regarded as an effective tool for resolving complicated evolutionary issues, such as genetic marker development, the genetic structure of the population, phylogenetic relations among species, and plant molecular identification. With the rapid progress of next-generation sequencing technologies, a growing count of complete plant cp DNA sequences are available in the National Center for Biotechnology Information (NCBI) database. 

*Symphytum officinale*, commonly known as comfrey, is a perennial herb belonging to the Boraginaceae that is widely distributed in Asia, Europe, and North America. This species was first introduced to China in 1963 and is now widely cultivated (Editorial Committee of Flora of China of Chinese Academy of Sciences, 1989). This plant is mainly found in damp habitats, particularly along rivers (Goulson *et al*., 1998). As a traditional medicine, comfrey has been widely used externally to treat bone fractures, tendon damage, strain, fatigue, and joint inflammation for over 2,000 years (Staiger, 2007; Salehi *et al*., 2019). Modern pharmacological research has shown that this plant contains a variety of substances, such as rosmarinic acid, chlorogenic acid, caffeic acid, and mucilage (Trifan *et al*., 2023). In addition, the plant has a rapid growth rate and can produce two to five crops per year (Bremness, 1988), making it a source of organic fertilizer, green manure, and animal feed (Wilkinson, 2003). However, some studies have indicated that the pyrrolizidine alkaloids contained in comfrey may cause hepatotoxicity in ruminant livestock (Dewick, 2002).

Although extensive research has been conducted on *S. officinale* owing to its medicinal value, ensilage (Wilkinson, 2003), breeding system, and pollination (Goulson *et al*., 1998), Insufficient attention has been directed towards inferring the phylogenetic position of *S. officinale* within Boraginaceae due to the scarcity of informative genetic markers, which has impeded the comprehension of the genetic diversity of Boraginaceae. Currently, approximately 56 whole cp genomes from the Boraginaceae have been deposited in the GenBank database (NCBI; http://www.ncbi.nlm.nih.gov/) Organelle Genome Resources; However, to date, no complete chloroplast (cp) genome sequences of *Symphytum* have been recorded in GenBank. In this study, we examined the complete plastid genome of *S. officinale* utilizing Illumina sequencing technology and compared its structure, gene arrangement, and IR borders with those of other publicly available plastid genomes from the Boraginaceae family*.* Our results provide genetic information on the cp of *S. officinale* to infer phylogenetic relations in Boraginaceae*.*

## Material and Methods

### Plant materials and DNA extraction

Fresh *S. officinale* leaves used in this study were collected from Xianyang (108°43′E, 34°15′N) in Shaanxi Province, China. A specimen voucher of the herbarium (SSX09422) has been archived at the Herbarium of Guizhou Education University. Genomic DNA was isolated via an adapted CTAB procedure (Doyle, 1987), the quantity and integrity of the DNA were assessed using a NanoDrop 2000 Spectrophotometer and electrophoresis in 1% (w/v) agarose gel. 

### Illumina sequencing, assembly, and annotation

The raw reads were obtained using an Illumina HiSeq X Ten sequencer, which provided an average read length of 150 base pairs for paired-end sequencing. Subsequently, the raw reads underwent quality trimming with the NGSQC Toolkit_v2.3.3 (Patel and Jain, 2012), employing the default threshold settings to eliminate potentially poor-quality bases. The assembly was conducted using the de novo method with GetOrganelle at default settings (Jin *et al*., 2020), and the resulting assembly graphs were inspected using Bandage (Wick *et al*., 2015) to verify the automatically determined plastomes. All genes were annotated employing Dual Organellar Genome Annotator software (Wyman *et al*., 2004) with default parameters. Subsequently, the GENEIOUS R8.0.2 program (Biomatters Ltd., Auckland, New Zealand) was used to correct the annotations based on comparisons with *Nonea vesicaria*, which was identified as the closest relative in the subsequent phylogenetic analysis. Circle maps of *S. officinale* were generated using Organellar Genome DRAW software (http://ogdraw.mpimp-golm.mpg.de) (Lohse *et al*., 2013). The complete cp genome of *S. officinale* has been submitted to GenBank (Accession Number: PQ645282).

### Screening coding sequences for codon usage bias analysis

In living organisms, every amino acid excluding methionine and tryptophan is specified by between two to six different synonymous codons (Guan *et al*., 2018). The selection of synonymous codons across various plant genomes is not arbitrary, this phenomenon is recognized as synonymous codon usage (SCU) bias (Wright, 1990). Understanding SCU bias not only illustrates mutations, but also helps to clarify the the origin and evolutionary history of species **(** Tuller *et al*., 2010; Quax *et al*., 2015).

In our research, we analyzed codon usage patterns in the *S. officinale* cp genome. Firstly, the protein-coding sequences of *S. officinale* were extracted manually according to the following criteria: (1) repeated sequences were eliminated; (2) CDSs with a length of 300 base pairs or less were not considered, as they tend to cause substantial errors in the estimation of codon usage; and (3) each CDS had to include a proper start codon (ATG) and termination codon (TAA, TAG, or TGA) (Li et al. 2016). Secondly, the extracted CDSs were used to calculate RSCU (relative synonymous codon usage) using CodonW v1.3 software (https://sourceforge.net/projects/codonw/).

### Sequence analysis and repeat structure

Besides *S. officinale,* we randomly selected 20 cp genomes (Table S1) belonging to four subfamilies (Boraginoideae, Cordioideae, Enteroideae, and Heliotropioideae) of the family Boraginaceae (Editorial Committee of Flora of China of Chinese Academy of Sciences, 1989). In total, multiple alignments of 21 Boraginaceae plastomes were performed using MAFFT v7.017 (Katoh and Standley, 2013). Full alignments of the 21 species were visualized using mVISTA software (Frazer *et al*., 2004). The cp DNA rearrangement analyses were performed using Mauve Alignment (Darling *et al*., 2004). Tandem repeat (TRs) sequences were identified using the online program Tandem Repeats Finder (Benson, 1999), with two, seven, and seven sets for the alignment parameters for match, mismatch, and indel, respectively. The minimum alignment score and maximum period were set to 80 and 500, respectively. Dispersed repeats (including forward, reverse, palindrome, and complement sequences) were determined using the program REPuter (Kurtz *et al*., 2001) (http://bibiserv.techfak.uni-bielefeld.de/reputer/manual.html) with a minimum repeat size of 30 bp and sequence identity greater than 90% (Hamming distance equal to three). The positions and types of simple sequence repeats (SSRs) were ascertained using the online software MISA (http://pgrc.ipk-gatersleben.de/misa/), with a minimum number of repeats of 10, five, four, three, three, and three for the mono-, di-, tri-, tetra-, penta-, and hexanucleotides, respectively. Nucleotide diversity of the chloroplast (cp) genome was analyzed using DnaSP v5.10 (Librado and Rozas, 2009), employing a sliding window method to assess genetic variation across the genome. The step size was set to 200 bp, with an 800 bp window length. Considering the inversions in *Cordia*, we removed two *Cordia* cp genomes when performing the nucleotide diversity analysis.

### Phylogenetic analysis 

To reveal the phylogenetic location of *S. officinale* and related species, 26 plastomes were included in this phylogenetic analysis, 21 of which were from Boraginaceae, with two species (*Justicia gendarussa* and *Justicia lianshanica*) from Acanthaceae and three species (*Leptoboea multiflora*, *Boeica multinervia*, and *Litostigma coriaceifolium*) from Gesneriaceae serving as outgroups (Table S1). Considering the existence of inversions in the *Cordia* cp genomes, phylogenetic analyses were performed utilizing 63 shared protein-coding genes among the 26 species. The datasets were aligned using MAFFT v7.402 (Katoh and Standley, 2013). Two phylogenetic trees were established employing the maximum likelihood (ML) and Bayesian inference (BI) methods. The best-fitting model for each dataset was determined using Modeltest 3.7 (Posada and Crandall, 1998) under the Akaike information criterion. ML analysis was conducted using RAxML v7.2.8 (Stamatakis, 2006) with 1,000 bootstrap replicates. BI was executed with MrBayes v3.12 (Ronquist and Huelsenbeck, 2003). The Markov chain Monte Carlo (MCMC) analysis was executed for 2,000,000 generations, with the initial 25% of the trees being discarded as burn-in. The consensus tree was then constructed from the remaining trees.

## Results

###  Cp genome of *S. officinale*

**Figure 1:**
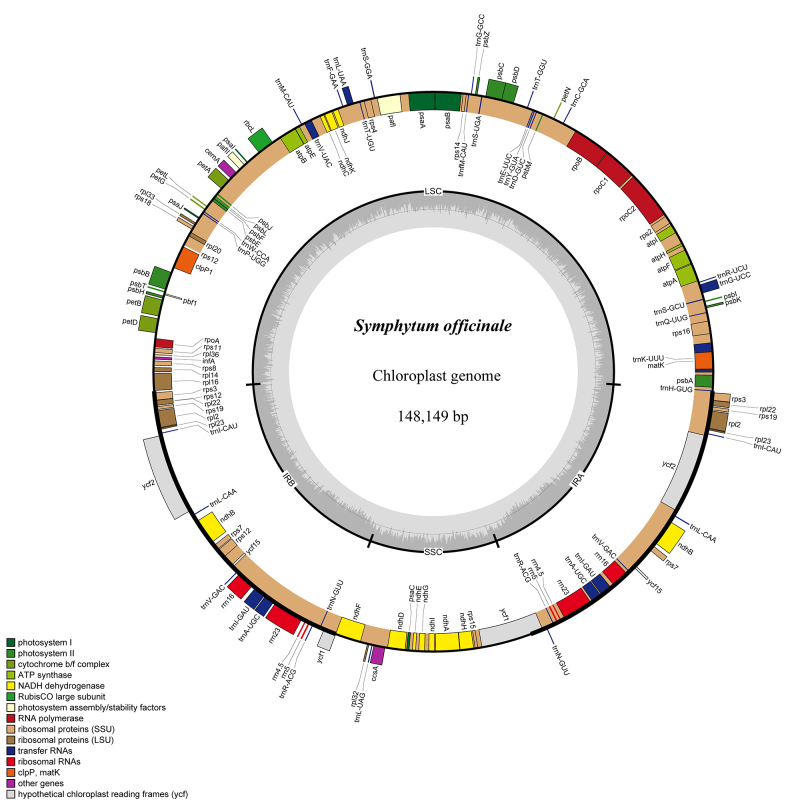
Gene maps of *S. officinale* chloroplast genomes. The genes shown outside of the circle are transcribed clockwise, while those inside are transcribed counterclockwise. Genes belonging to different functional groups are color coded. Dashed area in the inner circle indicates the GC content of the chloroplast genome.

In total, 28,274,782 paired-end reads with an average length of 150 bp were obtained. Following the assembly of the chloroplast (cp) genome, the complete cp genome of *S. officinale* was determined to be 148,149 bp in length. It exhibited the typical quadripartite structure found in angiosperms, comprising a large single-copy (LSC) region of 77,366 bp, a small single-copy (SSC) region of 16,781 bp, and two inverted repeats (IRa and IRb), each spanning 27,001 bp (Figure 1). The *S. officinale* cp genome encodes 133 predicted functional genes, which include 86 protein-coding genes, 37 tRNA genes, eight rRNA genes, and two pseudogenes. Among these genes, nine protein-coding genes (*rpl2, rpl22, rps3, rps7, rps12, rps19, ndhB, ycf1,* and *ycf2*), seven tRNA genes (*trnA-UGC, trnI-CAU, trnI-GAU, trnL-CAA, trnN-GUU, trnR-ACG,* and *trnV-GAC*), and four rRNA genes (*rrn4.5, rrn5, rrn16,* and *rrn23*) were duplicated in the IR regions. The *rpl23* gene, with two copies, was identified as a pseudogene (Table 1). The GC content of the *S. officinale* cpDNA was determined to be 38.0% overall. In the LSC, SSC, and IR regions, the GC content values were 36.0%, 31.7%, and 42.8%, respectively. Additionally, 18 genes containing introns were identified within the *S. officinale* cp genome, including nine protein-coding genes (*rpl2, rpl16, rps16, rpoC1, ndhA, ndhB, petB, petD,* and *atpF*) and six tRNA genes (*trnK-UUU, trnA-UGC, trnG-UCC, trnI-GAU, trnL-UAA,* and *trnV-UAC*) with one intron, three protein-coding genes (*rps12, clpP,* and *ycf3*) had two introns (Table 2). The *trnK-UUU* gene, which contained the largest intron of 2,454 bp, housed the *matK* gene within its sequence.

**Table 1:** Genes in the sequenced *S. officinale* chloroplast genome.

Category	Group of genes	Name of genes
Self-replication	ribosomal Proteins (Large subunit) (10)	rpl2^*^(×2), rpl14, rpl16^*^, rpl20, rpl22(×2), rpl32, rpl33, rpl36
	ribosomal proteins (Small subunit) (16)	rps2, rps3(×2), rps4, rps7(×2), rps8, rps11, rps12^**^(×2), rps14, rps15, rps16^*^, rps18, rps19(×2)
	RNA polymerase (4)	rpoA, rpoB, rpoC1^*^, rpoC2
	rRNA genes (8)	rrn4.5(×2), rrn5(×2), rrn16(×2), rrn23(×2)
	tRNA genes (37)	trnA-UGC^*^(×2), trnC-GCA, trnD-GUC, trnE-UUC, trnF-GAA, trnfM-CAU, trnG-GCC, trnG-UCC^*^, trnH-GUG, trnI-CAU(×2), trnI-GAU(×2)^*^, trnK-UUU^*^, trnL-CAA(×2), trnL-UAA^*^, trnL-UAG, trnM-CAU, trnN-GUU(×2), trnP-UGG, trnQ-UUG, trnR-ACG(×2), trnR-UCU, trnS-GCU, trnS-GGA, trnS-UGA, trnT-GGU, trnT-UGU, trnV-GAC(×2), trnV-UAC^*^, trnW-CCA, trnY-GUA
Photosynthesis	Photosystem I (5)	psaA, psaB, psaC, psaI, psaJ
	Photosystem II (15)	psbA, psbB, psbC, psbD, psbE, psbF, psbH, psbI, psbJ, psbK, psbL, psbM, psbN, psbT, psbZ
	NADH dehydrogenase (12)	ndhA^*^, ndhB^*^(×2), ndhC, ndhD, ndhE, ndhF, ndhG, ndhH, ndhI, ndhJ, ndhK
	Cytochrome b6/f complex (6)	petA, petB^*^, petD^*^, petG, petL, petN
	ATP synthase (6)	atpA, atpB, atpE, atpF^*^ , atpH, atpI
	Rubisco large subunit (1)	rbcL
Other genes	Maturase (1)	matK
	ATP-dependent protease subunit (1)	clpP^**^
	Membrane protein (1)	cemA
	c-type Cytochrome biogenesis (1)	ccsA
	Translation-related gene (1)	infA
Pseudogenes	ribosomal Proteins (Large subunit) (2)	rpl23(2)
Unknown	Conserved Open reading frames (6)	ycf1(×2), ycf2(×2), ycf3^**^, ycf4

*Gene contains one intron; ** gene contains two introns; (×2) indicates the number of the repeat unit is 2.

**Table 2:** Genes with introns in the chloroplast genome of *S. officinale*.

Gene	Location(bp)	Exon I(bp)	Intron I(bp)	Exon II (bp)	Intron II(bp)	Exon III(bp)
trnK-UUU	LSC	35	2454	37		
rps16	LSC	212	848	40		
trnG-UCC	LSC	23	687	48		
atpF	LSC	145	707	410		
rpoC1	LSC	432	763	1617		
ycf3	LSC	124	743	230	724	153
trnL-UAA	LSC	35	465	50		
trnV-UAC	LSC	38	605	35		
rps12^∗^	LSC/IRb	232	535	26	27387	114
clpP	LSC	71	596	292	763	228
petB	LSC	6	771	642		
petD	LSC	8	743	475		
rpl16	LSC	9	1084	399		
rpl2	IR	391	664	434		
ndhB	IR	777	670	756		
trnI-GAU	IR	37	947	35		
trnA-UGC	IR	38	812	35		
ndhA	SSC	553	986	539		

*rps12 gene is trans-spliced gene with the two duplicated 3’ end exons in IR regions and 5’ end exon in the LSC region.

### Codon use preference analysis

**Figure 2:**
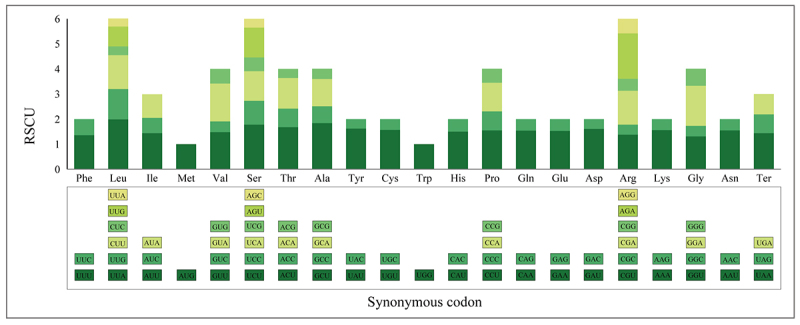
Summary of codon usage of cp genome of *S. officinale.*

Finally, 52 CDSs in the *S. officinale* cp genome were screened for codon usage pattern analysis with 20,685 codons (including 52 termination codons), according to the criteria mentioned in the previous chapters (Table S2). There were 61 types of codons (excluding stop codons) encoding 20 amino acids. Of these codons, the highest number was AUU, which encodes Isoleucine (858; 4.15%), and the lowest number was UGC, which encodes cysteine (47; 0.23%). Relative SCU (RSCU) is significant measure for assessing codon usage bias (Sharp and Li, 1986) and refers to the observed frequency of a codon divided by the expected frequency. An RSCU value of one indicates that synonymous codons are utilized at equal rates, while values above one suggest a preference for certain codons, and values below one imply a lower than average usage of those codons (Gupta *et al*., 2004). Our results indicated that 30 codons exhibited RSCU values surpassing one, with 29 of these being A or U-ending, signifying a bias towards A/T-ending codons. The leucine-specific codon UUA was particularly favored, showing an RSCU value of 1.99. Conversely, 32 codons had RSCU values below one, with 29 ending in C or G. The CUG codon, encoding leucine, had the lowest RSCU value at 0.32. Codons for methionine (AUG) and tryptophan (UGG) had RSCU values equal to one, suggesting no bias in their usage (Figure 2).

### Repeat analysis

**Figure 3:**
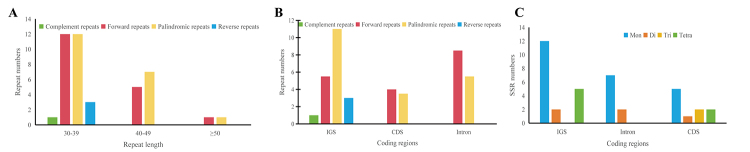
Repeat sequences and SSR in *S. officinale.* A. Frequency of Repeat sequences by Length; B. Frequency of Repeat sequences in the IGS, Intron, and CDS; C. Frequency of SSRs in the IGS, Intron, and CDS.

Six TRs were detected in the *S. officinale* cp genome (Table S3). The length of TRs ranged from 18 to 28 bp, and all TRs were distributed in the IR region, of which four were located in intergenic spacer (IGS) regions and two were in protein-coding (CDS) regions. Forty-two dispersed repeats, including 18 forward repeats, 20 palindromic repeats, three reverse repeats, and one complement repeat, were detected in the *S. officinale* cp genome (Table S4). Most repeats were located in the LSC region. The lengths of most repeats (66.67%) were 30-39 bp, followed by 40-49 bp (28.57%) and greater than 50 bp (4.76%) (Figure 3A); 48.81% of repeats in the *S. officinale* cp genome were distributed in IGSs, 33.33% were found in introns, and 17.86% in CDS (Figure 3B). A sum of 38 SSRs were identified in the *S. officinale* cp genome, the majority of which were mononucleotide repeats (24), followed by tetranucleotides (seven), dinucleotides (five), and trinucleotides (two); no pentanucleotide or hexanucleotide repeats were found (Table S5). All mononucleotide and dinucleotide repeats were composed of A or T, and only four SSRs in trinucleotide and tetranucleotide SSRs contained C or G bases. 50% of all SSRs were detected in IGSs, 26.32% were in CDSs, and 23.68% were in introns (Figure 3C). Similarly, the LSC region contained the most SSRs.

### IR contraction and expansion in the *S. officinale* cp genome 

**Figure 4:**
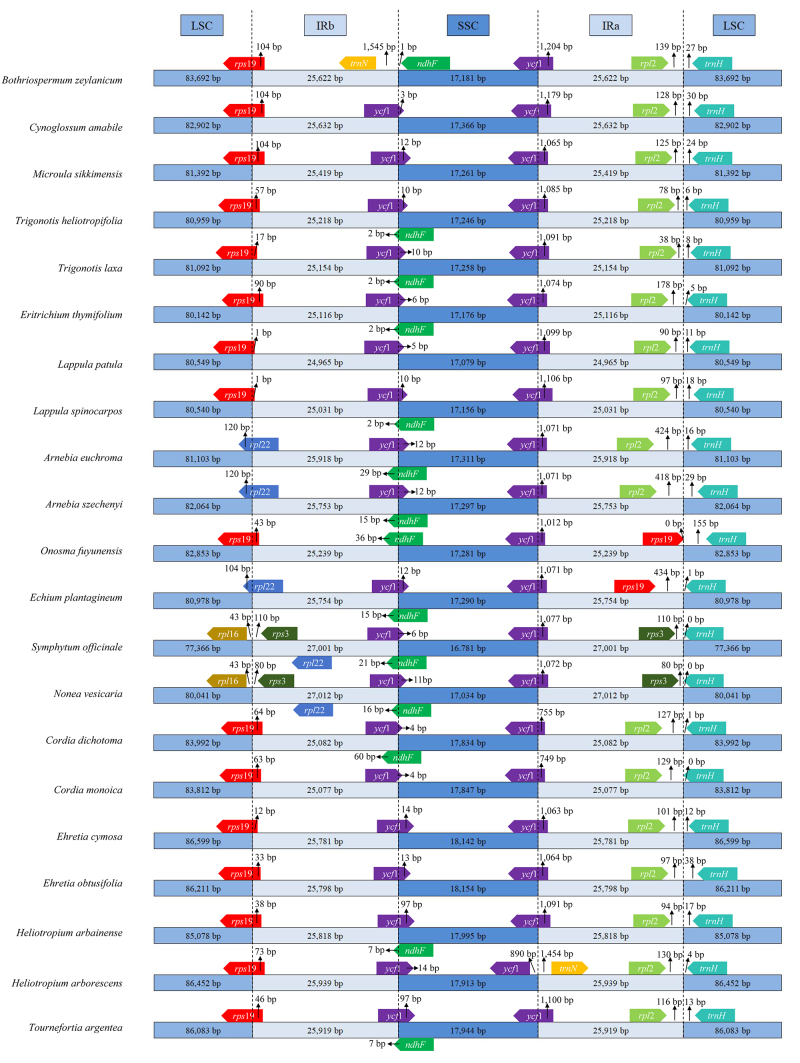
Comparison of IR, LSC and SSC region borders among *S. officinale* and its related species in Boraginaceae.

The IR/LSC and IR/SSC borders of 21 Boraginaceae cp genomes were compared. Considerable variations were noted regarding the expansion and contraction of the IR regions. Nine genes (*rps19*, *ycf1*, *trnN-GUU*, *ndhF*, *rpl2*, *trnH*, *rpl22*, *rpl16*, and *rps3*) were observed at the LSC/IR and SSC/IR borders of the 21 plastomes. The genomic structure, including gene number and gene order, of *S. officinale* was similar to that of *Nonea vesicaria*. However, genes located at the SC/IR borders in the two species differed from those of other plastomes. Figure 4 shows that *S. officinale* and *N. vesicaria* both exhibited larger plastome sizes (27,001-27,012 bp) than those of the other 19 Boraginaceae plastomes (24,965-25,939 bp) because of increased IR length. Correspondingly, the SCs of *S. officinale* and *N. vesicaria* (LSC: 77,366-80,041 bp; SSC: 16,781-17,034 bp) were shorter than those of other cp genomes (LSC: 80,142-86,599 bp; SSC: 17,079-18,154 bp). In *S. officinale* and *N. vesicaria*, genes located at the LSC/IRb border were *rpl16 and rps3*, which were situated entirely in the LSC and IRb, respectively. The *rpl22* gene crossed the LSC/IRb border in three species (*Arnebia euchroma*, *Arnebia szechenyi*, and *Echium plantagineum*), whereas the *rps19* gene crossed the LSC/IRb border in the remaining 16 species. The *ycf1* gene crossed the IRb/SSC border in seven species, the *ndhF* gene crossed the IRb/SSC border in one species, and the *ycf1* gene in the IRb region and *ndhF* gene in the SSC region interlaced at the IRb/SSC border in 12 species; however, in *Bothriospermum zeylanicum*, *trnN* and *ndhF* were located entirely in the IRb and SSC, respectively. The *ycf1* gene crossed the SSC/IRa region in all species except *Heliotropium arborescens*. In this species, *ycf1* and *trnN* were located entirely in the SSC and IRa respectively. At the LSC/IRa border, the gene in the LSC was always *trnH*, but the gene in the IRa varied: *rps3* in *S. officinale* and *N. vesicaria*, *rps19* in *Onosma fuyunensis* and *E. plantagineum*, and *rpl2* in the remaining 17 species.

### Comparisons of cp DNA of 21 Boraginaceae species

**Figure 5:**
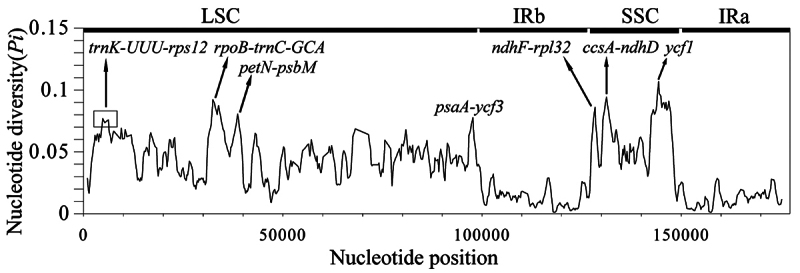
Comparison of nucleotide diversity (*Pi*) values among 19 Boraginaceae species.

To understand the level of sequence divergence, mVISTA was employed for a comparative analysis of the cp genome sequences among 21 species of Boraginaceae, utilizing *N. vesicaria* (Leonardo *et al*., 2022) as a reference. The comparison demonstrated highly conserved coding regions among the 21 species compared to non-coding regions (Figure S1). That is, the regions exhibiting the highest divergence across the 21 cp genomes were identified within the IGS. The cp genome of *Cordia* revealed an inversion of the *trnM-rbcL* region compared to the genomes of other species, leading to the observed gene rearrangements in the LSC region (Figure S2). The sliding window analysis revealed the same tendency of genetic variation of the chloroplast genomes of these 19 Boraginaceae species. Specifically, the IR regions showed lower divergence in comparison to the large LSC and SSC regions (Table S6, Figure 5). All highly variable regions were located in the SC region, including *trnK-UUU-rps12*, *rpoB-trnC-GCA*, *petN-psbM*, *psaA-ycf3*, *ndhF-rpl32*, *ccsA-ndhD*, and *ycf1*. 

### Phylogenetic analyses based on Cp genome sequence 

**Figure 6:**
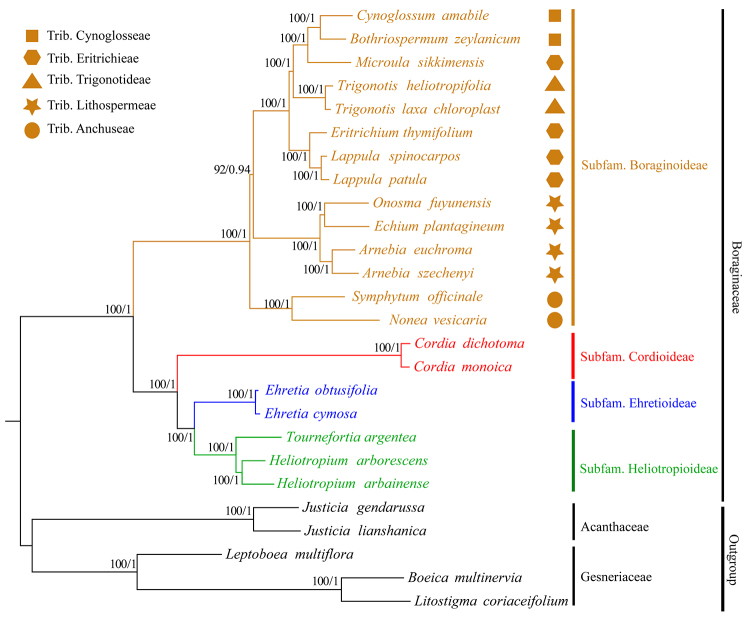
Phylogenetic relationships of the 26 species inferred from ML and BI analyses based on CDS regions. Numbers above the lines on the left indicate the ML bootstrap of clade >50%, numbers above the lines on the right indicate the Bayesian posterior probabilities.

To investigate the phylogenetic position of *S. officinale* within the Boraginaceae family, we analyzed 63 protein-coding genes that are shared among the chloroplast genomes of 26 members, representing 21 Boraginaceae species and five relatives from Acanthaceae and Gesneriaceae as outgroups (Table S1, Figure 6). The best ML and BI tree models were the GTR+I+G and TVM+I+G, respectively. The phylogenetic trees constructed using the ML and Bayesian methods had identical topological structures, although the support values differed slightly. All 21 species from the Boraginaceae formed a monophyletic group with high bootstrap and BI support. Furthermore, species in each subfamily formed a single clade. *S. officinale* is closely related to *N. vesicaria*, and these two species constitute the earliest diverging lineage in this subfam. Boraginoideae. Most species clustered together according to morphological classification, except *Microula sikkimensis*, which appeared to be more closely related to Trib. Cynoglosae species than to Trib. Eritrichieae species.

## Discussion

In the present study, we determined the complete plastid genome sequence of *S. officinale*, which is the first complete plastome of *Symphytum*, using Illumina sequencing technology. Subsequently, a comparative examination of genome structure, codon usage, sequence variation, and nucleotide diversity were conducted with its relatives in the Boraginaceae. The results showed that the plastome of *S. officinale* had structural features similar to those observed in other angiosperms, with a LSC, a SSC region, and two IR regions (Chen and Zhang, 2019; Li and Wei, 2022; Sun *et al*., 2022; Wu *et al*., 2022; Alawfi and Albokhari, 2023; Alshegaihi *et al*., 2023; Yan *et al*., 2023; Noroozi *et al*., 2024). Our results show that the IRs were more conserved than the SC regions, which may be related to copy correction between IR sequences via gene conversion (Khakhlova and Bock, 2006). In addition, the GC content in the IR region was higher than in the SC regions, which could be associated with the presence of rRNA genes, characterized by high GC content, in the IR region (Liu *et al*., 2021). The majority of genes commonly found in the plastomes of angiosperms are present in the *S. officinale* plastome. However, *rpl23* has degenerated into a pseudogene in this plastome and has also been reported as a pseudogene in the cp genome of *Platycodon grandiflorus* (Zhang *et al*., 2023). The function of the pseudogene *rpl23* in spinach is replaced by a gene in the nucleus (Bubunenko *et al*., 1994). Chiefari *et al* (2010) indicated that pseudogenes play a significant role in maintaining the stable expression of functional genes. Therefore, studying pseudogenes is important for further understanding the structure and evolution of the genome. Inversions have been identified in cp genomes of many angiosperms., eg. *Lasthenia burkei* (Walker *et al*., 2014), *Euphrasia regelii* (Zhou *et al*., 2019), the inversion of the *trnM-rbcL* region in the cp genome of *Cordia* has been reported in detail by scholars(Alawfi and Albokhari, 2023), which may be associated with intramolecular recombination events in GC-rich regions or tRNA activity within the genome (Hiratsuka *et al*., 1989; Walker *et al*., 2014).

In this study, *rpl16*/*rps3* was a boundary gene located in the LSC/IRb region in *S. officinale* and *N. vesicaria*, but the boundary genes of the other 19 species were *rps19* or *rpl22*, which were located in the IRb region in *S. officinale* and *N. vesicaria,* demonstrating an expansion of the IR regions in these species*.* Contraction and expansion of the IR boundary region are usually observed in plants, which is considered a primary evolutionary mechanism (Kode *et al*., 2005; Raubeson *et al*., 2007; Yao *et al*., 2015).

Repeats occur across the plastomes of all land plants (Provan *et al*., 2001; George *et al*., 2015). In this study, we found six TRs and 42 dispersed repeats in the *S. officinale* plastid genome. As reported for other genomes (Bausher *et al*., 2006; Jansen *et al*., 2006; Ruhlman *et al*., 2006; Ni *et al*., 2016), A multitude of repeats within the *S. officinale* plastid genome are situated in the IGS, while a minor portion of these repeats are found in the CDS region, potentially a result of the cp genome expansion. Plastid genetic markers with high polymorphism are usually considered the main source of structural variation in plastomes and can be used in population genetics studies (Powell *et al*., 1995; Patel and Jain, 2012; Rogalski *et al*., 2008; Pauwels *et al.,* 2012). Meanwhile, we found 38 SSRs in the *S. officinale* plastid genome. Among these SSRs, the highly abundant mononucleotides and dinucleotides were mostly composed of A/T repeats, suggesting that the SSRs in the cp genome of *S. officinale* were more abundant in A/T than in G/C repeats, similar to those screened in other plants (Huang *et al*., 2014; Wheeler *et al*., 2014; George *et al*., 2015; Cauz-Santos *et al*., 2017). Compared with CG, AT has only two hydrogen bond connections, making it more prone to breakage. The replication process is prone to unstable phenomena, such as recombination and slippage, which could increase the probability of microsatellite formation to some extent (Borsch and Quandt, 2009). Additionally, SSRs are associated with genomic recombination and rearrangement (Cavalier-Smith, 2002).

Complete cp genome sequences are suitable for the phylogenetic analysis of Boraginaceae (Sun *et al*., 2022; Noroozi *et al*., 2024). In addition, individual cp genomes have been reported, enriching the genetic information on Boraginaceae (Chen and Zhang, 2019; Li and Wei, 2022; Wu et al., 2022; Alawfi and Albokhari, 2023; Alshegaihi et al., 2023; Wu et al., 2022; Yan et al., 2023). In the present study, the 63 shared protein-coding genes from 26 plants, including 21 species from Boraginaceae and five individuals from Acanthaceae and Gesneriaceae, were employed to construct phylogenetic trees via ML and BI, which indicated that the 21 ingroups represented four subfamilies in Boraginaceae, consistent with the classification in the *Flora Reipublicae Popularis Sinicae* (Editorial Committee of Flora of China of Chinese Academy of Sciences, 1989). As individuals belonging to the Trib. Anchuseae, *S. officinale* and *N. vesicaria* were grouped into a monophyletic clade located at the base of the subfam. Boraginoideae, suggesting that Trib. Anchuseae may have diverged early in the subfamily. Boraginoideae. As a member of Trib. Eritrichieae, *M. sikkimensis* clustered into Trib. Cynoglosseae species. Therefore, the classification of *M. sikkimensis* should emphasize how this species is genetically unique compared to its other relatives. In short, both the ML and BI phylogenetic results in the current study were strongly supported by 100% bootstrap values, but this is still incomplete due to the limited taxonomic sampling available. Further phylogenetic studies are necessary based on morphological characteristics, nuclear genomes, and cp genome datasets. Expanded taxon sampling is required to acquire an accurate relation in this group.

## Conclusion

In this study, we sequenced the cp genome of *S. officinale* and conducted a comparative analysis with 20 other published cp genomes within the Boraginaceae. Gene order and genome organization in the *S. officinale* cp genome are similar to other Boraginaceae cp genomes. A comparative analysis of the 21 species in Boraginaceae revealed seven highly variable regions. Six TRs, 42 dispersed repeats, and 38 SSRs were identified. These findings could be used for population genetic studies of *Symphytum.* Phylogenetic analysis demonstrated that *S. officinale* is closely related to *N. vesicaria*. This marks the first cp genome to be sequenced for *Symphytum*, thus contributing significant genetic insights for molecular discrimination and evolutionary studies.

## Data availability

The genome sequence data that support the findings of this study are available in GenBank under the accession PQ645282. The associated BioProject, SRA, and BioSample numbers are PRJNA1189664, SRR31484011, and SAMN45006900, respectively.

## Supplementary material

The following online material is available for this article:

Table S1 - The features of chloroplast genomes of 26 species used for phylogenetic inference in this study

Table S2 - RSCU analysis of protein coding region in the chloroplast of *S. officinale*

Table S3 - List of tandem repeats in the chloroplast genome of *S. officinale*

Table S4 - List of dispersed repeats in the chloroplast genome of *S. officinale*

Table S5 - List of SSR in the chloroplast genome of *S. officinale*

Table S6 - DNA polymorphism of the chloroplast genomes of 19 Boraginaceae species

Figure S1 - Sequence identity plot comparing the 21 Boraginaceae chloroplast genomes with *Nonea vesicaria* as a reference by using mVISTA

Figure S2 - MAUVE alignment of 21 Boraginaceae species chloroplast genomes

## References

[B1] Alawfi MS, Albokhari EJ (2023). Comparative chloroplast genomics reveals a unique gene inversion
in two cordia trees (Cordiaceae). Forests.

[B2] Alshegaihi RM, Mansour H, Alrobaish SA, Shaye NAA, El-Moneim DA (2023). The first complete chloroplast genome of Cordia monoica:
Structure and comparative analysis. Genes.

[B3] Bausher MG, Singh ND, Lee SB, Jansen RK, Daniell H (2006). The complete chloroplast genome sequence of Citrus sinensis (L.)
Osbeck var ‘ridge pineapple’: Organization and phylogenetic relationships to
other angiosperms. BMC Plant Biol.

[B4] Benson G (1999). Tandem repeats finder: A program to analyze DNA
sequences. Nucleic Acids Res.

[B5] Borsch T, Quandt D (2009). Mutational dynamics and phylogenetic utility of noncoding
chloroplast DNA. Plant Syst Evol.

[B6] Bremness L (1988). The Complete Book of Herbs.

[B7] Bubunenko MG, Schmidt J, Subramanian AR (1994). Protein substitution in chloroplast ribosome evolution: A
eukaryotic cytosolic protein has replaced its organelle homologue (L23) in
spinach. J Mol Biol.

[B8] Cauz-Santos LA, Munhoz CF, Rodde N, Cauet S, Santos AA, Penha HA, Dornelas MC, Varani AM, Oliveira GCX, Bergès H (2017). The chloroplast genome of Passifora edulis (Passiforaceae)
assembled from long sequence reads: Structural organization and phylogenomic
studies in Malpighiales. Front Plant Sci.

[B9] Cavalier-Smith T (2002). Chloroplast evolution: Secondary symbiogenesis and multiple
losses. Curr Biol.

[B10] Chen Q, Zhang D (2019). The complete chloroplast genome sequence of Onosma paniculatum
Bur. et Franch. (Boraginaceae), a medicinal plant in Yunnan and its adjacent
regions. Mitochondrial DNA B Resour.

[B11] Chiefari E, Iiritano S, Paonessa F, Pera IL, Arcidiacono B, Filocamo M, Foti D, Liebhaber SA, Brunetti A (2010). Nat Commun.

[B12] Darling AC, Mau B, Blattner FR, Perna NT (2004). Mauve: Multiple alignment of conserved genomic sequence with
rearrangements. Genome Res.

[B13] Dewick PM (2002). Medicinal Natural Products: A Biosynthetic Approach.

[B14] Doyle JJ (1987). A rapid DNA isolation procedure for small quantities of fresh
leaf tissue. Phytochem Bull.

[B15] Editorial Committee of Flora of China of Chinese Academy of
Sciences (1989). Flora Reipublicae Popularis Sinicae.

[B16] Frazer KA, Pachter L, Poliakov A, Rubin EM, Dubchak I (2004). VISTA: Computational tools for comparative
genomics. Nucleic Acids Res.

[B17] George B, Bhatt BS, Awasthi M, George B, Singh AK (2015). Comparative analysis of microsatellites in chloroplast genomes of
lower and higher plants. Curr Genet.

[B18] Goulson D, Stout JC, Hawson SA, Allen JA (1998). Floral display size in comfrey, Symphytum officinale L.
(Boraginaceae): Relationships with visitation by three bumblebee species and
subsequent seed set. Oecologia.

[B19] Guan DL, Ma LB, Khan MS, Zhang XX, Xu SQ, Xie JY (2018). Analysis of codon usage patterns in Hirudinaria manillensis
reveals a preference for GC-ending codons caused by dominant selection
constraints. BMC Genomics.

[B20] Gupta SK, Bhattacharyya TK, Ghosh TC (2004). Synonymous codon usage in Lactococcus lactis, mutational bias
versus translational selection. J Biomol Struct Dyn.

[B21] Hiratsuka J, Shimada H, Whittier R, Ishibashi T, Sakamoto M, Mori M, Kondo C, Honji Y, Sun CR, Meng BY (1989). The complete sequence of the rice (Oryza sativa) chloroplast
genome: Intermolecular recombination between distinct transfer RNA genes
accounts for a major plastid DNA inversion during the evolution of the
cereals. Mol Gen Genet.

[B22] Huang H, Shi C, Liu Y, Mao SY, Gao LZ (2014). Thirteen Camellia chloroplast genome sequences determined by
high-throughput sequencing: genome structure and phylogenetic
relationships. BMC Evol Biol.

[B23] Inoue K (2011). Emerging roles of the chloroplast outer envelope
membrane. Trends Plant Sci.

[B24] Jansen RK, Kaittanis C, Saski C, Lee SB, Tomkins J, Alverson AJ, Daniell H (2006). Phylogenetic analyses of Vitis (Vitaceae) based on complete
chloroplast genome sequences: Effects of taxon sampling and phylogenetic
methods on resolving relationships among rosids. BMC Evol Biol.

[B25] Jin JJ, Yu WB, Yang JB, Song Y, de Pamphilis CW, Yi TS, Li DZ (2020). GetOrganelle: A fast and versatile toolkit for accurate de novo
assembly of organelle genomes. Genome Biol.

[B26] Katoh K, Standley DM (2013). MAFFT Multiple sequence alignment software version 7:
Improvements in performance and usability. Mol Biol Evol.

[B27] Khakhlova O, Bock R (2006). Elimination of deleterious mutations in plastid genomes by gene
conversion. Plant J.

[B28] Kode V, Mudd EA, Iamtham S, Day A (2005). The tobacco plastid accD gene is essential and is required for
leaf development. Plant J.

[B29] Kurtz S, Choudhuri JV, Ohlebusch E, Schleiermacher C, Stoye J, Giegerich R (2001). REPuter: The manifold applications of repeat analysis on a
genomic scale. Nucleic Acids Res.

[B30] Leonardo IC, Barreto Crespo MT, Capelo J, Gaspar FB (2022). The complete plastome of Nonea vesicaria (L.) Rchb.
(Boraginaceae), the first chloroplast genome belonging to the Nonea
genus. Mitochondrial DNA B Resour.

[B31] Li N, Li YY, Zheng CC, Huang JG, Zhang SZ (2016). Genome-wide comparative analysis of the codon usage patterns in
plants. Genes Genom.

[B32] Li Q, Wei R (2022). Comparison of Boraginales plastomes: Insights into codon usage
bias, adaptive evolution, and phylogenetic relationships. Diversity.

[B33] Librado P, Rozas J (2009). Dnasp v5: A software for comprehensive analysis of DNA
polymorphism data. Bioinformatics.

[B34] Liu K, Wang R, Guo XX, Zhang XJ, Qu XJ, Fan SJ (2021). Comparative and phylogenetic analysis of complete chloroplast
genomes in eragrostideae (Chloridoideae, Poaceae). Plants.

[B35] Lohse M, Drechsel O, Kahlau S, Bock R (2013). OrganellarGenomeDRAW-A suite of tools for generating physical
maps of plastid and mitochondrial genomes and visualizing expression data
sets.. Nucleic Acids Res.

[B36] Ni LH, Zhao ZL, Dorje G, Ma M (2016). The complete chloroplast genome of Ye-Xing-Ba (Scrophularia
dentata; Scrophulariaceae), an alpine Tibetan herb. PLoS One.

[B37] Noroozi M, Ghahremaninejad F, Riahi M, Cohen JI (2024). Phylogenomics and plastome evolution of Lithospermeae
(Boraginaceae). BMC Plant Biol.

[B38] Patel RK, Jain M (2012). NGS QC Toolkit: A toolkit for quality control of next generation
sequencing data. PLoS One.

[B39] Pauwels M, Vekemans X, Gode C, Frerot H, Castric V, SaumitouLaprade P (2012). Nuclear and chloroplast DNA phylogeography reveals vicariance
among European populations of the model species for the study of metal
tolerance, Arabidopsis halleri (Brassicaceae). New Phytol.

[B40] Provan J, Powell W, Hollingsworth PM (2001). Chloroplast microsatellites: New tools for studies in plant
ecology and evolution. Trends Ecol Evol.

[B41] Powell W, Morgante M, Mcdevitt R, Vendramin GG, Rafalski JA (1995). Polymorphic simple sequence repeat regions in chloroplast
genomes-applications to the population-genetics of pines. Proc Natl Acad Sci USA.

[B42] Posada D, Crandall KA (1998). Modeltest: Testing the model of DNA substitution. Bioinformatics.

[B43] Quax TE, Claassens NJ, Söll D, Van der Oost J (2015). Codon bias as a means to fine-tune gene
expression. Mol Cell.

[B44] Raubeson LA, Peery R, Chumley TW, Dziubek C, Fourcade HM, Boore JL, Jansen RK (2007). Comparative chloroplast genomics: Analyses including new
sequences from the angiosperms Nuphar advena and Ranunculus
macranthus. BMC Genomics.

[B45] Rogalski M, Schottler MA, Thiele W, Schulze WX, Bock R (2008). Rpl33, a nonessential plastid-encoded ribosomal protein in
tobacco, is required under cold stress conditions. Plant Cell.

[B46] Ronquist F, Huelsenbeck JP (2003). Mrbayes 3: Bayesian phylogenetic inference under mixed
models. Bioinformatics.

[B47] Ruhlman T, Lee SB, Jansen RK, Hostetler JB, Tallon LJ, Town CD, Daniell H (2006). Complete plastid genome sequence of Daucus carota: Implications
for biotechnology and phylogeny of angiosperms. BMC Genomics.

[B48] Salehi B, Sharopov F, Tumer TB, Ozleyen A, Rodríguez-Pérez C, Ezzat SM, Azzini E, Hosseinabadi T, Butnariu M, Sarac I (2019). Symphytum Species: A comprehensive review on chemical
composition, food applications and phytopharmacology. Molecules.

[B49] Sharp PM, Li WH (1986). An evolutionary perspective on synonymous codon usage in
unicellular organisms. J Mol Evol.

[B50] Staiger C (2007). Comfrey: Ancient and modern uses. Pharm J.

[B51] Stamatakis A (2006). RAxML-VI-HPC: Maximum likelihood-based phylogenetic analysis with
thousands of taxa and mixed models. Bioinformatics.

[B52] Sun J, Wang S, Wang Y, Wang R, Liu K, Li E, Qiao P, Shi L, Dong W, Huang L (2022). Phylogenomics and genetic diversity of Arnebiae radix and its
allies (Arnebia, Boraginaceae) in China. Front Plant Sci.

[B53] Trifan A, Czerwińska ME, Zengin G, Esslinger N, Grubelnik A, Wolfram E, Skalicka-Woźniak K, Luca SV (2023). Influence of pyrrolizidine alkaloids depletion upon the
biological activity of Symphytum officinale L. extracts. J Ethnopharmacol.

[B54] Tuller T, Waldman YY, Kupiec M, Ruppin E (2010). Translation efficiency is determined by both codon bias and
folding energy. Proc Natl Acad Sci USA.

[B55] Walker JF, Zanis MJ, Emery NC (2014). Comparative analysis of complete chloroplast genome sequence and
inversion variation in Lasthenia burkei (Madieae,
Asteraceae). Am J Bot.

[B56] Wheeler GL, Dorman HE, Buchanan A, Challagundla L, Wallace LE (2014). A review of the prevalence, utility, and caveats of using
chloroplast simple sequence repeats for studies of plant
biology. Appl Plant Sci.

[B57] Wick RR, Schultz MB, Zobel J, Holt KE (2015). Bandage: Interactive visualization of de novo genome
assemblies. Bioinformatics.

[B58] Wilkinson JM (2003). A laboratory evaluation of comfrey (Symphytum officinale L.) as a
forage crop for ensilage. Anim Feed Sci Technol.

[B59] Wright F (1990). The ‘effective number of codons’ used in a gene. Gene.

[B60] Wu J, Li H, Lei J, Liang Z (2022). The complete chloroplast genome sequence of Trigonotis
peduncularis (Boraginaceae). Mitochondrial DNA B Resour.

[B61] Wyman SK, Jansen RK, Boore JL (2004). Automatic annotation of organellar genomes with
DOGMA. Bioinformatics.

[B62] Yan S, Wang T, Wang Z, Ren W, Liu C, Ma W, Dong S (2023). The chloroplast genome of Lappula myosotis V. Wolf, a medicinal
species. Mitochondrial DNA B Resour.

[B63] Yang M, Zhang X, Liu G, Yin Y, Chen K, Yun Q, Zhao D, Al-Mssallem IS, Yu J (2010). The complete chloroplast genome sequence of date palm (Phoenix
dactylifera L.). PLoS One.

[B64] Yao XH, Tang P, Li ZZ, Li DW, Liu YF, Huang HW (2015). The first complete chloroplast genome sequences in Actinidiaceae:
Genome structure and comparative analysis. PLoS One.

[B65] Zhang X, Zhou T, Kanwal N, Zhao Y, Bai G, Zhao G (2017). Completion of eight Gynostemma bl. (Cucurbitaceae) chloroplast
genomes: Characterization, comparative analysis, and phylogenetic
relationships. Front Plant Sci.

[B66] Zhang Y, Du C, Zhang H, Shang C, Li R, Yuan S (2023). Comparative and phylogeny analysis of Platycodon grandiflorus
complete chloroplast genomes. Chin Tradit Herbal Drugs.

[B67] Zhou T, Ruhsam M, Wang J, Zhu H, Li W, Zhang X, Xu Y, Xu F, Wang X (2019). The complete chloroplast genome of Euphrasia regii,
pseudogenization of ndh genes and the phylogenetic relationships within
Orobanchaceae. Front Genet.

