# Supplementary material for: Complete chloroplast genome sequence and phylogenetic analysis of Symphytum officinale
Source: Genet Mol Biol. 2025 Jun 30;48(2):e20240258. doi: 10.1590/1678-4685-GMB-2024-0258 (PMC12210358; doi:10.1590/1678-4685-GMB-2024-0258)
Supplement: Table S1 - [file 1415-4757-GMB-48-2-e20240258-s1.pdf]

Genetics and Molecular Biology
https://doi.org/10.1590/1678-4685-GMB-2024-0258

Supplementary Material to: Complete chloroplast genome sequence and phylogenetic analysis of *Symphytum officinale*

Table S1-The features of chloroplast genomes of 26 species used for phylogenetic inference in this study

No.	Species	Family	Genbank accession number	Size (bp)	LSC (bp)	SSC (bp)	IR (bp)	Number of Protein-Coding Genes	Number of tRNA Genes	Number of rRNA Genes	GC Content (%)	Pseudogene
1	*Bothriospermum zeylanicum*	Boraginaceae	NC_065834	152,117	83,692	17,181	25,622	84(6)	37(7)	8(4)	37.4	0
2	*Cynoglossum amabile*	Boraginaceae	NC_061706	151,532	82,902	17,366	25,632	84(5)	37(7)	8(4)	37.4	1
3	*Microula sikkimensis*	Boraginaceae	NC_085483	149,428	81,329	17,261	25,419	84(6)	37(7)	8(4)	37.5	1
4	*Trigonotis heliotropifolia*	Boraginaceae	NC_070030	148,641	80,959	17,246	25,218	85(6)	37(7)	8(4)	37.6	0
5	*Trigonotis laxa*	Boraginaceae	NC_070031	148,658	81,092	17,258	25,154	85(6)	37(7)	8(4)	37.6	0
6	*Eritrichium thymifolium*	Boraginaceae	ON550364	147,550	80,142	17,176	25,116	85(6)	37(7)	8(4)	37.7	0
7	*Lappula patula*	Boraginaceae	NC_077514	147,558	80,549	17,079	24,965	85(6)	37(7)	8(4)	37.7	1
8	*Lappula spinocarpos*	Boraginaceae	NC_077512	147,758	80,540	17,156	25,031	85(6)	37(7)	8(4)	37.7	1
9	*Arnebia euchroma*	Boraginaceae	NC_053782	150,250	81,103	17,311	25,918	86(7)	37(7)	8(4)	37.6	1
10	*Arnebia szechenyi*	Boraginaceae	NC_066702	150,867	82,064	17,297	25,753	86(6)	37(7)	8(4)	37.7	1
11	*Onosma*	Boraginaceae	NC_049569	150,612	82,853	17,281	25,239	84(5)	37(7)	8(4)	37.5	4

No.	Species	Family	Genbank accession number	Size (bp)	LSC (bp)	SSC (bp)	IR (bp)	Number of Protein-Coding Genes	Number of tRNA Genes	Number of rRNA Genes	GC Content (%)	Pseudogene
	fuyunensis											
12	*Echium plantagineum*	Boraginaceae	NC_067370	149,776	80,978	17,290	25,754	86(6)	37(7)	8(4)	37.5	0
13	*Symphytum officinale*	Boraginaceae	PQ645282	148,149	77,366	16,781	27,001	86(8)	37(7)	8(4)	38	2
14	*Nonea vesicaria*	Boraginaceae	NC_060826	151,099	80,041	17,034	27,012	89(9)	37(7)	8(4)	37.3	0
15	*Cordia dichotoma*	Boraginaceae	NC_066967	151,990	83,992	17,834	25,082	82(6)	37(7)	8(4)	38.2	0
16	*Cordia monoica*	Boraginaceae	OP793888	151,813	83,812	17,847	25,077	87(7)	37(7)	8(4)	38.2	0
17	*Ehretia cymosa*	Boraginaceae	NC_084199	156,328	86,599	18,142	25,781	88(7)	37(7)	8(4)	37.9	0
18	*Ehretia obtusifolia*	Boraginaceae	NC_084108	155,961	86,211	18,154	25,798	88(7)	37(7)	8(4)	37.9	0
19	*Heliotropium arbainense*	Boraginaceae	NC_084200	154,709	85,078	17,995	25,818	88(7)	37(7)	8(4)	37.7	0
20	*Heliotropium arborescens*	Boraginaceae	NC_066966	156,243	86,452	17,913	25,939	83(7)	37(7)	8(4)	37.7	0
21	*Tournefortia argentea*	Boraginaceae	NC_073098	155,865	86,083	17,944	25,919	87(7)	37(7)	8(4)	37.9	1
22	*Justicia gendarussa*	Acanthaceae	NC_080241	149,735	82,373	17,218	25,072	86(6)	37(7)	8(4)	38	2
23	*Justicia lianshanica*	Acanthaceae	NC_080243	148,574	81,775	16,880	24,958/24,911	86(6)	37(7)	8(4)	38	2
24	*Leptoboea multiflora*	Gesneriaceae	NC_070430	154,089	85,195	17,920	25,487	81(6)	37(7)	8(4)	38	0
25	*Boeica*	Gesneriaceae	NC_070432	154,058	85,219	17,995	25,422	86(7)	36(7)	8(4)	38	0

No.	Species	Family	Genbank accession number	Size (bp)	LSC (bp)	SSC (bp)	IR (bp)	Number of Protein-Coding Genes	Number of tRNA Genes	Number of rRNA Genes	GC Content (%)	Pseudogene
	multinervia											
26	*Litostigma coriaceifolium*	Gesneriaceae	NC_070433	153,788	85081	17907	25,400	85(7)	37(7)	8(4)	37.4	0

Numbers in brackets denote the numbers of genes duplicated in the IR regions.
