# Supplementary material for: Complete chloroplast genome sequence and phylogenetic analysis of Symphytum officinale
Source: Genet Mol Biol. 2025 Jun 30;48(2):e20240258. doi: 10.1590/1678-4685-GMB-2024-0258 (PMC12210358; doi:10.1590/1678-4685-GMB-2024-0258)
Supplement: Table S2 - [file 1415-4757-GMB-48-2-e20240258-s2.pdf]

Genetics and Molecular Biology
https://doi.org/10.1590/1678-4685-GMB-2024-0258

Supplementary Material to: Complete chloroplast genome sequence and phylogenetic analysis of *Symphytum officinale*

Table S2 - RSCU analysis of protein coding region in the chloroplast of *S. officinale*

AA	Codon	Number	RSCU	AA	Codon	Number	RSCU	AA	Codon	Number	RSCU
Phe	UUU	785	1.36	Thr	ACU	447	1.68	Glu	GAA	832	1.53
	UUC	372	0.64		ACC	197	0.74		GAG	259	0.47
Leu	UUA	730	1.99		ACA	323	1.22	Asp	GAU	658	1.61
	UUG	446	1.21		ACG	96	0.36		GAC	161	0.39
	CUU	495	1.35	Ala	GCU	533	1.84	Arg	CGU	283	1.38
	CUC	128	0.35		GCC	194	0.67		CGC	83	0.40
	CUA	290	0.79		GCA	316	1.09		CGA	277	1.35
	CUG	116	0.32		GCG	118	0.41		CGG	99	0.48
Ile	AUU	858	1.44	Tyr	UAU	624	1.62		AGA	371	1.81
	AUC	365	0.61		UAC	148	0.38		AGG	118	0. 58
	AUA	561	0.94	Cys	UGU	173	1.57	Lys	AAA	817	1.56
Met	AUG	474	1.00		UGC	47	0.43		AAG	233	0.44
Val	GUU	419	1.48	Trp	UGG	387	1.00	Gly	GGU	477	1.31
	GUC	123	0.43	His	CAU	372	1.50		GGC	151	0.42
	GUA	427	1.51		CAC	123	0.50		GGA	580	1.60
	GUG	164	0.58	Pro	CCU	340	1.55		GGG	246	0.68
Ser	UCU	448	1.78		CCC	166	0.76	Asn	AAU	753	1.55
	UCC	239	0.95		CCA	251	1.14		AAC	221	0.45
	UCA	297	1.18		CCG	122	0.56	TER	UAA	25	1.44
	UCG	139	0.55	Gln	CAA	595	1.54		UAG	13	0.75
	AGU	299	1.19		CAG	178	0.46		UGA	14	0.81
	AGC	89	0.35								

AA: Amino acid
