# Supplementary material for: Complete chloroplast genome sequence and phylogenetic analysis of Symphytum officinale
Source: Genet Mol Biol. 2025 Jun 30;48(2):e20240258. doi: 10.1590/1678-4685-GMB-2024-0258 (PMC12210358; doi:10.1590/1678-4685-GMB-2024-0258)
Supplement: Table S3 - [file 1415-4757-GMB-48-2-e20240258-s3.pdf]

Genetics and Molecular Biology
https://doi.org/10.1590/1678-4685-GMB-2024-0258

Supplementary Material to: Complete chloroplast genome sequence and phylogenetic analysis of *Symphytum officinale*

Table S3 - List of tandem repeats in the chloroplast genome of *S. officinale*.

No.	Indice		Copy number	Consensus Size	Percent Matches	Percent Indels	size	Repeat sequence	Region
1	81080	81128	2.5	20	100	0	20	TTAGGAGAAATCAATGCCAT	IRb;IGS;(*trnI-CAU-ycf*2)
2	81052	81105	2	27	92	7	28	TTAGGAGAAATCAATGCGCCATTTAGGA	IRb;IGS;(*trnI-CAU-ycf*2)
3	86145	86204	3.3	18	97	0	18	CGATATTGATGCTAGTGA	IRb;CDS;*ycf*2
4	139312	139371	3.3	18	97	0	18	ATATCGTCACTAGCATCG	IRa;CDS;*ycf*2
5	144388	144436	2.5	20	100	0	20	TTCTCCTAAATGGCATTGAT	IRa;IGS(*ycf*2-*trnI-CAU*)
6	144411	144464	2	28	92	7	28	TCCTAAATGGCATTGATTTCTCCTAATC	IRa;IGS(*ycf*2-*trnI-CAU*)
