# Supplementary material for: Complete chloroplast genome sequence and phylogenetic analysis of Symphytum officinale
Source: Genet Mol Biol. 2025 Jun 30;48(2):e20240258. doi: 10.1590/1678-4685-GMB-2024-0258 (PMC12210358; doi:10.1590/1678-4685-GMB-2024-0258)
Supplement: Table S4 - [file 1415-4757-GMB-48-2-e20240258-s4.pdf]

Genetics and Molecular Biology
https://doi.org/10.1590/1678-4685-GMB-2024-0258

Supplementary Material to: Complete chloroplast genome sequence and phylogenetic analysis of *Symphytum officinale*

Table S4 - List of dispersed repeats in the chloroplast genome of *S. officinale*

Repeat Type	No.	Length of the 1st repeat	Starting position of the 1st repeat	Length of the 2nd repeat	Starting position of the 2nd repeat	Mismatch	E-value	Repeat sequences	RegionA	RegionB
C	1	31	93842	31	131642	-2	5.60E-06	CGAGGAGAAGGGGGCTCGGCGGGAAGAGGA	IRb;IGS(rps12-trnV-GAC)	IRa;IGS(trnV-GAC-rps12)
F	1	52	37660	52	39884	-2	3.63E-18	GAGAAAAAGAATTGCAATAGCTAAATGATGATGAGCCATATCAGTCAGCCAT	LSC;CDS(*psaB*)	LSC;CDS(*psaA*)
F	2	46	86144	46	86162	-1	1.72E-16	TCGATATTGATGCTAGTGACGATATTGATGCTAGTGACGATATCGA	IRb;CDS(*ycf*2)	IRb;CDS(*ycf*2)
F	3	46	139307	46	139325	-1	1.72E-16	GATCGATATCGTCACTAGCATCGATATCGTCACTAGCATCAATATC	IRa;CDS(*ycf*2)	IRa;CDS(*ycf*2)
F	4	39	92858	39	113506	-1	2.39E-12	ACAGAACCGTACATGAGATTTTCACCTCATACGGCTCCT	IRb; IGS(*rps*12-*trnV*-*GAC*)	SSC;intron(*ndhA*)

Repeat Type	No.	Length of the 1st repeat	Starting position of the 1st repeat	Length of the 2nd repeat	Starting position of the 2nd repeat	Mismatch	E-value	Repeat sequences	RegionA	RegionB
F	5	48	76268	48	92858	-2	3.58E-11	TCAGAACCGGACATGAGATTTTCACCTCATCCGGCTCCTCGCGACGAA	LSC;intron(*rpl*16)	IRb; IGS(*rps*12-*trnV*-*GAC*)
F	6	42	42635	42	113503	-3	9.89E-11	GTTTCAGAACCGTACGTGAGATTTTCATCTCATACGGCTCCT	LSC;intron(*ycf*3)	SSC;intron(*ndhA*)
F	7	39	42638	39	92858	-2	1.36E-10	TCAGAACCGTACGTGAGATTTTCATCTCATACGGCTCCT	LSC;intron(*ycf*3)	IRb; IGS(*rps*12-*trnV*-*GAC*)
F	8	41	76266	41	113504	-3	3.67E-10	CTTCAGAACCGGACATGAGATTTTCACCTCATCCGGCTCCT	LSC;intron(*rpl*16)	SSC;intron(*ndhA*)
F	9	31	76277	31	92867	-1	1.24E-07	GACATGAGATTTTCACCTCATCCGGCTCCTC	LSC;intron(*rpl*16)	IRb; IGS(*rps*12-*trnV*-*GAC*)
F	10	30	81078	30	81098	-1	4.82E-07	GATTAGGAGAAATCAATGCCATTTAGGAGA	IRb; IGS(*trnI-CAU-ycf2*)	IRb; IGS(*trnI-CAU-ycf*2)
F	11	30	144386	30	144406	-1	4.82E-07	TCTTCTCCTAAATGGCATTGATTTCTCCTA	IRa; IGS(*cyf*2-*trnI-CAU*)	IRa; IGS(*ycf2*-*trnI-CAU*)

Repeat Type	No.	Length of the 1st repeat	Starting position of the 1st repeat	Length of the 2nd repeat	Starting position of the 2nd repeat	Mismatch	E-value	Repeat sequences	RegionA	RegionB
F	12	35	89811	35	113509	-3	9.24E-07	AAACCGTGCATGAGACTTTCATCTCACACGGCTCC	IRb;intron(*ndhB*)	SSC;intron(*ndhA*)
F	13	30	76277	30	113515	-2	2.10E-05	GACATGAGATTTTCACCTCATCCGGCTCCT	LSC;intron(*rpl*16)	SSC;intron(*ndhA*)
F	14	32	7786	32	34517	-3	4.48E-05	GAAACGGAAAGAGAGGGATTCGAACCCTCGGT	LSC; IGS(*psbI*-*trnS*-*GCU*)	LSC; IGS(psbC-*trnS*-*UGA*)
F	15	31	42636	31	76266	-3	1.62E-04	TTTCAGAACCGTACGTGAGATTTTCATCTCA	LSC;intron(*ycf*3)	LSC;intron(*rpl*16)
F	16	30	9064	30	35482	-3	5.87E-04	AACGATGCGGGTTCGATTCCCGCTACCCGC	LSC;CDS(*trnG*-*UCC*)	LSC;CDS(*trnG*-*GCC*)
F	17	36	42647	36	76277	-3	5.87E-04	TACGTGAGATTTTCATCTCATACGGCTCCTCCCTTA	LSC;intron(*ycf*3)	LSC;intron(*rpl*16)
F	18	30	42650	30	92870	-3	5.87E-04	GTGAGATTTTCATCTCATACGGCTCCTCCC	LSC;intron(*ycf*3)	IRb; IGS(*rps*12-*trnV*-*GAC*)
P	1	70	25929	70	76507	-2	9.63E-29	ATTGATATGTTTGCACAAATAATTGAAATTCGATTTCTTGATCTGTATCTTCAAT	LSC;CDS(*rpoB*)	LSC;intron(*rpl*16)

Repeat Type	No.	Length of the 1st repeat	Starting position of the 1st repeat	Length of the 2nd repeat	Starting position of the 2nd repeat	Mismatch	E-value	Repeat sequences	RegionA	RegionB
								TTTTGGAAACTTATA		
P	2	44	68981	44	68981	0	1.99E-17	ATTGAAGTAATAAGCCTCCCAATATTGGGAGGCTTATTACTTCA	LSC; IGS(*psbT*-*psbN*)	LSC; IGS(*psbT*-*psbN*)
P	3	44	108732	44	108732	0	1.99E-17	CGAACCGTGTGAATCAATACAATATTGTATTGATTCACACGGTT	SSC; IGS(ccsA-*ndhD*)	SSC; IGS(ccsA-*ndhD*)
P	4	46	25953	46	76507	-1	1.72E-16	GAAATTCGATTTCTTGATCTGTATCTTCAATTTTTGGAAACTTATA	LSC;CDS(*rpoB*)	LSC;intron(*rpl*16)
P	5	46	86144	46	139307	-1	1.72E-16	TCGATATTGATGCTAGTGACGATATTGATGCTAGTGACGATATCGA	IRb;CDS(*ycf*2)	IRa;CDS(*ycf*2)
P	6	46	86162	46	139325	-1	1.72E-16	ACGATATTGATGCTAGTGACGATATCGATGCTAGTGACGATATCGA	IRb;CDS(*ycf*2)	IRa;CDS(*ycf*2)
P	7	39	113506	39	132618	-1	2.39E-12	TCAGAACCGTACATGAGACTTTCACCTCATACGGCTCCT	SSC;intron(*ndhA*)	IRa; IGS(trnV-GAC-*rps7*)

Repeat Type	No.	Length of the 1st repeat	Starting position of the 1st repeat	Length of the 2nd repeat	Starting position of the 2nd repeat	Mismatch	E-value	Repeat sequences	RegionA	RegionB
P	8	48	76268	48	132617	-2	3.58E-11	TCAGAACCGGACA TGAGATTTTCACCT CATCCGGCTCCTC GCGACGAA	LSC;intron(*rpl*16)	IRa; IGS(trnV-GAC-*rps7*)
P	9	40	110384	40	110384	-2	3.58E-11	CACAAAACCCGTG CTCAAAATAATTT GAGCACGGGTTTT G	SSC;IGS(*ndhD-psaC*)	SSC;IGS(*ndhD-psaC*)
P	10	39	42638	39	132618	-2	1.36E-10	TCAGAACCGTACG TGAGATTTTCATCT CATACGGCTCCT	LSC;intron(*ycf*3)	IRa; IGS(trnV-GAC-*rps7*)
P	11	37	13561	37	13561	-3	6.86E-08	ATAGTTGCTTTTGG TCAATTCTTGACC AAAAGCAACT	LSC; IGS(*atpH-atpI*)	LSC; IGS(*atpH-atpI*)
P	12	31	76277	31	132617	-1	1.24E-07	GACATGAGATTTT CACCTCATCCGGC TCCTC	LSC;intron(*rpl*16)	IRa; IGS(trnV-GAC-*rps7*)
P	13	30	7788	30	43684	-1	4.82E-07	AACGGAAAGAGA GGGATTCGAACCC TCGGT	LSC; IGS(psbI-*trnS-GCU*)	LSC;CDS(*trnS-GGA*)
P	14	30	81078	30	144387	-1	4.82E-07	GATTAGGAGAAAT CAATGCCATTTAG GAGA	IRb; IGS(*trnI-CAU-ycf*2)	IRa; IGS(*ycf*2-*trnI-CAU*)
P	15	30	81098	30	144407	-1	4.82E-07	ATTTAGGAGAAAT CAATGCCATTTAG	IRb; IGS(*trnI-CAU-ycf*	IRa; IGS(*ycf*2-*trnI-CAU*)

Repeat Type	No.	Length of the 1st repeat	Starting position of the 1st repeat	Length of the 2nd repeat	Starting position of the 2nd repeat	Mismatch	E-value	Repeat sequences	RegionA	RegionB
								GAGA	2)	
P	16	35	113509	35	135669	-3	9.24E-07	GAACCGTACATGAGACTTTCACCTCATACGGCTCC	SSC;intron(*ndhA*)	IRa;intron*ndhB*)
P	17	32	44874	32	44891	-3	4.48E-05	TATTCTTTGATTTCAAAGTTTCAATCATTGAA	LSC; IGS(*rps4*-*trnT-UGU*)	LSC; IGS(*rps4*-*trnT-UGU*)
P	18	32	71592	32	71612	-3	4.48E-05	TGTATTTATTCCTCTAAAAAAAGATTCCAATC	LSC;intron(*petD*)	LSC;intron(*petD*)
P	19	31	6738	31	6738	-3	1.62E-04	GAATATTAATAATATTTAATATTATTAGTAT	LSC; IGS(trnQ-UUG-*psbK*)	LSC; IGS(trnQ-UUG-*psbK*)
P	20	30	42650	30	132615	-3	5.87E-04	GTGAGATTTTCATCTCATACGGCTCCTCCC	LSC;intron(*ycf*3)	IRa; IGS(trnV-GAC-*rps7*)
R	1	31	43765	31	43765	-2	5.60E-06	CTTTCTTTGATATAGAAAGATATAGATTCTT	LSC; IGS(*trnS-GGA*-*rps*4)	LSC; IGS(*trnS-GGA*-*rps*4)
R	2	31	93842	31	93842	-2	5.60E-06	CGAGGAGAAGGGGGCTCGGCGGGAAGAGGA	IRb; IGS(*rps*12-*trnV-GAC*)	IRb; IGS(*rps*12-*trnV-GAC*)
R	3	31	131642	31	131642	-2	5.60E-06	ACTCCTCTTCCCGCCGAGCCCCCTTCTCCT	IRa; IGS(trnV-GAC-*rps7*)	IRa; IGS(trnV-GAC-*rps7*)

C: Complement repeats; F: Forward repeats; P: Palindromic repeats; R: Reverse repeats
