# Supplementary material for: Complete chloroplast genome sequence and phylogenetic analysis of Symphytum officinale
Source: Genet Mol Biol. 2025 Jun 30;48(2):e20240258. doi: 10.1590/1678-4685-GMB-2024-0258 (PMC12210358; doi:10.1590/1678-4685-GMB-2024-0258)
Supplement: Table S5 - [file 1415-4757-GMB-48-2-e20240258-s5.pdf]

Genetics and Molecular Biology
https://doi.org/10.1590/1678-4685-GMB-2024-0258

Supplementary Material to: Complete chloroplast genome sequence and phylogenetic analysis of *Symphytum officinale*

Table S5 - List of SSR in the chloroplast genome of *S. officinale*.

No.	SSR type	SSR	Size	Start	End	SSR type		
1	p1	(A)10	10	13899	13908	LSC	IGS	*atpH-atpI*
2	p1	(A)10	10	22161	22170	LSC	intron	*rpoC1*
3	p1	(A)10	10	113799	113808	SSC	intron	*ndhA*
4	p1	(A)11	11	12633	12643	LSC	IGS	*atpF-atpH*
5	p1	(A)11	11	30861	30871	LSC	IGS	*trnT-GGU-psbD*
6	p1	(A)11	11	65495	65505	LSC	intron	*clpP*
7	p1	(A)11	11	65973	65983	LSC	intron	*clpP*
8	p1	(A)12	12	59100	59111	LSC	IGS	*petA-psbJ*
9	p1	(A)13	13	12931	12943	LSC	IGS	*atpH-atpI*
10	p1	(A)14	14	5264	5277	LSC	intron	*rps16*
11	p1	(T)10	10	42342	42351	LSC	intron	*ycf3*
12	p1	(T)10	10	74741	74750	LSC	IGS	*infA-rps8*
13	p1	(T)10	10	106560	106569	SSC	IGS	*ndhF-rpl32*
14	p1	(T)10	10	107017	107026	SSC	IGS	*ndhF-rpl32*
15	p1	(T)11	11	7529	7539	LSC	IGS	*psbK-psbl*
16	p1	(T)11	11	69832	69842	LSC	intron	*petB*
17	p1	(T)11	11	72836	72846	LSC	IGS	*petD-rpoA*
18	p1	(T)11	11	116340	116350	SSC	CDS	*rps15*
19	p1	(T)11	11	117730	117740	SSC	CDS	*ycf1*
20	p1	(T)11	11	117988	117998	SSC	CDS	*ycf1*
21	p1	(T)12	12	29271	29282	LSC	IGS	*psbM-trnD-GUC*
22	p1	(T)12	12	119682	119693	SSC	CDS	*ycf1*
23	p1	(T)14	14	15689	15702	LSC	IGS	*rps2-rpoC2*
24	p1	(T)14	14	17749	17762	LSC	CDS	*rpoC2*
25	p2	(AT)5	10	30518	30527	LSC	IGS	*trnE-UUC-trnT-GGU*
26	p2	(AT)5	10	79583	79592	IRb	intron	*rpl2*
27	p2	(AT)5	10	118192	118201	SSC	CDS	*ycf1*
28	p2	(AT)5	10	145924	145933	IRa	intron	*rpl2*
29	p2	(TA)5	10	6085	6094	LSC	IGS	*rps16-trnQ-UUG*
30	p3	(AAG)4	12	2751	2762	LSC	CDS	*matK*
31	p3	(TTC)4	12	34152	34163	LSC	CDS	*psbC*
32	p4	(AATA)3	12	66906	66917	LSC	IGS	*clpP-psbB*
33	p4	(AATA)3	12	109059	109070	SSC	CDS	*ndhD*
34	p4	(ATAA)3	12	62775	62786	LSC	IGS	*rpl33-rps18*

No.	SSR type	SSR	Size	Start	End	SSR type		
35	p4	(ATAA)3	12	65774	65785	LSC	CDS	*clpP*
36	p4	(ATTT)3	12	116643	116654	SSC	IGS	*rps15-ycf1*
37	p4	(CTAT)3	12	34717	34728	LSC	IGS	*trnS-UGA-psbZ*
38	p4	(TCTA)3	12	40884	40895	LSC	IGS	*psaA-ycf3*
