# Supplementary material for: Complete chloroplast genome sequence and phylogenetic analysis of Symphytum officinale
Source: Genet Mol Biol. 2025 Jun 30;48(2):e20240258. doi: 10.1590/1678-4685-GMB-2024-0258 (PMC12210358; doi:10.1590/1678-4685-GMB-2024-0258)
Supplement: Table S6 - [file 1415-4757-GMB-48-2-e20240258-s6.pdf]

Genetics and Molecular Biology
https://doi.org/10.1590/1678-4685-GMB-2024-0258

Supplementary Material to: Complete chloroplast genome sequence and phylogenetic analysis of *Symphytum officinale*

Table S6 - DNA polymorphism of the chloroplast genomes of 19 Boraginaceae species

No.	Window/bp	Midpoint /bp	Pi	No.	Window/bp	Midpoint/ bp	Pi	No.	Window /bp	Midpoint/ bp	Pi
1	1-1151	851	0.03368	214	63452-64099	63764	0.02371	427	122572-123180	122879	0.00399
2	752-1351	1051	0.01776	215	63665-64317	63964	0.02352	428	122780-123387	123080	0.00497
3	952-1551	1251	0.01688	216	63865-64526	64204	0.02449	429	122980-123587	123287	0.00361
4	1152-1751	1451	0.01733	217	64100-64733	64417	0.02307	430	123181-123787	123487	0.0026
5	1352-2061	1651	0.02727	218	64318-64963	64626	0.02525	431	123388-123990	123687	0.00379
6	1552-2452	1863	0.03683	219	64527-65310	64842	0.03673	432	123588-124190	123887	0.00498
7	1752-2670	2173	0.05353	220	64734-65729	65083	0.05573	433	123788-124390	124090	0.00484
8	2062-2870	2570	0.05669	221	64964-66025	65442	0.0574	434	123991-124605	124290	0.00382
9	2453-3082	2770	0.05881	222	65311-66296	65875	0.04394	435	124191-124867	124490	0.00922
10	2671-3288	2976	0.06261	223	65730-66508	66172	0.02894	436	124391-125124	124706	0.01498
11	2871-3509	3188	0.0616	224	66026-66728	66396	0.02617	437	124606-125436	125024	0.02147
12	3083-3740	3397	0.06456	225	66297-66937	66628	0.03286	438	124868-125766	125268	0.02806
13	3289-3950	3609	0.06012	226	66509-67149	66831	0.029	439	125125-126105	125565	0.02674
14	3510-4228	3850	0.0613	227	66729-67360	67037	0.03215	440	125437-126358	125952	0.02478
15	3741-4496	4072	0.06356	228	66938-67564	67260	0.03646	441	125767-126611	126230	0.01526
16	3951-4759	4387	0.06072	229	67150-68183	67460	0.05657	442	126106-126814	126480	0.01273
17	4229-5292	4652	0.07302	230	67361-71456	67808	0.07018	443	126359-127014	126711	0.01177
18	4497-6164	4875	0.0778	231	67565-71981	71335	0.07432	444	126612-127214	126914	0.01339
19	4760-6368	5412	0.07948	232	68184-72181	71583	0.0585	445	126815-127507	127114	0.0202
20	5293-6679	6268	0.07128	233	71457-72381	72081	0.04352	446	127015-127773	127323	0.04062

21	6165-6992	6480	0.05979	234	71982-72587	72281	0.03248	447	127215-128191	127633	0.06975
22	6369-7279	6865	0.06314	235	72182-73900	72487	0.04043	448	127508-128394	128091	0.08107
23	6680-7543	7152	0.05463	236	72382-74100	73800	0.04165	449	127774-128618	128291	0.08375
24	6993-9240	7410	0.06725	237	72588-74300	74000	0.04269	450	128192-128875	128518	0.08021
25	7280-9486	9046	0.06532	238	73901-74544	74200	0.03555	451	128395-129075	128757	0.07373
26	7544-9707	9370	0.06745	239	74101-74779	74400	0.03406	452	128619-129275	128975	0.05346
27	9241-10175	9607	0.06187	240	74301-74979	74679	0.02882	453	128876-129475	129175	0.03591
28	9487-10421	9970	0.05898	241	74545-75179	74879	0.03217	454	129076-129675	129375	0.03887
29	9708-10847	10285	0.06567	242	74780-75385	75079	0.03725	455	129276-129875	129575	0.04137
30	10176-11739	10544	0.06567	243	74980-75585	75279	0.0384	456	129476-130075	129775	0.03955
31	10422-11990	11530	0.06439	244	75180-76638	75485	0.04499	457	129676-130296	129975	0.03448
32	10848-12211	11848	0.06262	245	75386-77002	75711	0.06331	458	129876-130675	130175	0.05743
33	11740-12457	12091	0.05995	246	75586-77222	76896	0.06587	459	130076-131246	130471	0.08852
34	11991-12823	12329	0.0577	247	76639-77430	77102	0.04835	460	130297-131792	130986	0.09737
35	12212-13140	12557	0.05258	248	77003-77630	77322	0.02017	461	130676-132632	131361	0.09176
36	12458-13340	12945	0.04092	249	77223-77833	77530	0.02045	462	131247-132868	132531	0.06576
37	12824-13540	13240	0.03519	250	77431-79029	77730	0.0399	463	131793-133074	132765	0.06446
38	13141-13740	13440	0.02948	251	77631-79284	77933	0.04897	464	132633-133277	132968	0.0493
39	13341-13940	13640	0.02582	252	77834-79623	79173	0.0575	465	132869-133492	133177	0.06051
40	13541-14140	13840	0.02885	253	79030-79888	79519	0.04645	466	133075-133692	133392	0.0582
41	13741-14340	14040	0.02709	254	79285-80239	79737	0.04209	467	133278-134137	133592	0.07322
42	13941-14540	14240	0.02809	255	79624-80668	80139	0.05634	468	133493-134337	133851	0.06269
43	14141-14783	14440	0.0263	256	79889-80912	80412	0.06022	469	133693-134537	134237	0.06152
44	14341-14983	14683	0.02842	257	80240-81277	80789	0.07282	470	134138-134737	134437	0.04327
45	14541-15215	14883	0.03706	258	80669-81500	81051	0.05788	471	134338-134937	134637	0.03622
46	14784-15515	15083	0.04907	259	80913-81854	81391	0.05786	472	134538-135137	134837	0.03825
47	14984-15754	15347	0.05767	260	81278-82054	81628	0.04093	473	134738-135337	135037	0.03772
48	15216-16007	15637	0.05049	261	81501-82330	81954	0.05519	474	134938-135537	135237	0.04951
49	15516-16387	15907	0.04895	262	81855-82553	82192	0.05405	475	135138-135758	135437	0.05152

50	15755-16632	16136	0.05193	263	82055-82753	82453	0.05945	476	135338-135958	135638	0.05384
51	16008-16832	16509	0.05335	264	82331-82968	82653	0.04179	477	135538-136165	135858	0.05136
52	16388-17857	16732	0.06308	265	82554-83254	82853	0.03991	478	135759-136413	136061	0.0448
53	16633-18309	17253	0.06607	266	82754-83499	83109	0.04339	479	135959-136634	136313	0.04488
54	16833-18509	18086	0.06218	267	82969-83754	83368	0.0483	480	136166-136962	136513	0.0473
55	17875-18709	18409	0.03997	268	83255-84023	83654	0.05462	481	136414-137162	136862	0.04316
56	18310-18909	18609	0.02715	269	83500-84286	83891	0.0549	482	136635-137362	137062	0.04225
57	18510-19144	18809	0.03375	270	83755-84551	84123	0.05371	483	136963-137860	137262	0.05322
58	18710-19540	19012	0.03892	271	84024-84815	84420	0.04848	484	137163-138060	137512	0.0572
59	18910-19740	19440	0.0383	272	84287-85041	84688	0.0452	485	137363-138260	137960	0.05849
60	19145-19940	19640	0.03335	273	84552-85246	84941	0.04044	486	137861-138485	138160	0.03862
61	19541-20166	19840	0.0354	274	84816-85552	85141	0.04379	487	138061-138700	138365	0.04075
62	19741-20568	20040	0.05085	275	85042-85869	85412	0.05118	488	138261-138900	138585	0.0418
63	19941-20768	20378	0.05745	276	85247-86155	85652	0.05693	489	138486-139151	138800	0.0449
64	20167-20968	20668	0.05077	277	85553-86462	86042	0.05408	490	138701-139599	139000	0.05081
65	20569-21168	20868	0.03397	278	85870-86729	86277	0.05917	491	138901-139829	139319	0.05735
66	20769-21368	21068	0.03565	279	86156-86957	86588	0.04797	492	139152-140144	139716	0.06546
67	20969-21580	21268	0.04942	280	86463-87157	86857	0.04138	493	139600-140403	139974	0.06144
68	21169-21804	21468	0.06313	281	86730-87357	87057	0.02654	494	139830-140603	140303	0.05938
69	21369-22023	21686	0.06331	282	86958-87557	87257	0.03065	495	140145-140803	140503	0.05117
70	21581-22223	21923	0.05596	283	87158-87760	87457	0.03402	496	140404-141013	140703	0.04498
71	21805-22429	22123	0.04857	284	87358-87960	87657	0.03495	497	140604-141222	140913	0.03991
72	22024-22632	22326	0.0543	285	87558-88160	87860	0.03272	498	140804-141422	141113	0.03745
73	22224-22838	22532	0.05622	286	87761-88407	88060	0.03395	499	141014-141622	141322	0.03945
74	22430-23065	22732	0.06391	287	87961-88696	88260	0.0493	500	141223-141822	141522	0.03529
75	22633-23265	22941	0.0592	288	88161-88925	88580	0.04746	501	141423-142022	141722	0.02648
76	22839-23465	23165	0.04692	289	88408-89135	88818	0.04428	502	141623-142257	141922	0.03097
77	23066-23665	23365	0.03378	290	88697-89374	89032	0.03221	503	141823-142534	142155	0.04382
78	23266-23871	23565	0.02946	291	88926-89610	89235	0.03723	504	142023-143040	142357	0.07933

79	23466-24071	23771	0.0395	292	89136-89940	89483	0.05256	505	142258-143291	142801	0.08779
80	23666-24271	23971	0.03389	293	89375-90257	89817	0.05535	506	142535-143563	143185	0.09083
81	23872-24471	24171	0.02788	294	89611-90464	90137	0.05036	507	143041-143766	143460	0.06667
82	24072-24684	24371	0.02456	295	89941-90664	90364	0.02996	508	143292-144056	143663	0.0832
83	24272-24918	24571	0.03609	296	90258-90864	90564	0.02284	509	143564-144313	143902	0.08798
84	24472-25121	24818	0.03946	297	90465-91106	90764	0.02765	510	143767-144531	144165	0.11189
85	24685-25330	25021	0.03739	298	90665-91332	90964	0.04802	511	144057-144907	144419	0.10011
86	24919-25530	25230	0.02802	299	90865-91694	91227	0.05161	512	144314-145128	144687	0.10036
87	25122-25730	25430	0.02732	300	91107-92086	91582	0.06324	513	144532-145417	145028	0.08634
88	25331-25930	25630	0.02741	301	91333-92286	91898	0.04583	514	144908-145692	145273	0.08378
89	25531-26130	25830	0.02884	302	91695-92486	92186	0.0412	515	145129-145925	145550	0.08965
90	25731-26330	26030	0.03159	303	92087-92712	92386	0.02934	516	145418-146176	145801	0.09356
91	25931-26575	26230	0.03142	304	92287-93325	92586	0.0469	517	145693-146385	146067	0.08511
92	26131-26875	26444	0.03929	305	92487-93531	93223	0.04984	518	145926-146630	146285	0.08456
93	26331-27125	26723	0.0434	306	92713-93731	93431	0.048	519	146177-146869	146485	0.08147
94	26576-27345	26993	0.04408	307	93326-93940	93631	0.03793	520	146386-147169	146769	0.09393
95	26876-27545	27236	0.03464	308	93532-94166	93840	0.03937	521	146631-147399	146990	0.08657
96	27126-27745	27445	0.0294	309	93732-94366	94050	0.04364	522	146870-147695	147290	0.08775
97	27346-27958	27645	0.0226	310	93941-94603	94266	0.04581	523	147170-148036	147595	0.07442
98	27546-28158	27858	0.02262	311	94167-94849	94479	0.04939	524	147400-148332	147927	0.05734
99	27749-28358	28058	0.01867	312	94367-95077	94703	0.04656	525	147696-148541	148217	0.03593
100	27959-28558	28258	0.02268	313	94604-95331	94964	0.04377	526	148037-148741	148441	0.01933
101	28159-28758	28458	0.02594	314	94850-95534	95210	0.03884	527	148333-148944	148641	0.01451
102	28359-28958	28658	0.0274	315	95078-95753	95431	0.04229	528	148542-149196	148844	0.01162
103	28559-29158	28858	0.02619	316	95332-96038	95634	0.03752	529	148742-149427	149055	0.0114
104	28759-29358	29058	0.02778	317	95535-96238	95938	0.03435	530	148945-149750	149327	0.01663
105	28959-29558	29258	0.02685	318	95754-96506	96138	0.03673	531	149197-150121	149566	0.02626
106	29159-29761	29458	0.0308	319	96039-96706	96389	0.04028	532	149428-150405	149937	0.02918
107	29359-29961	29661	0.02665	320	96239-96926	96606	0.04501	533	149751-150680	150264	0.0234

108	29559-30161	29861	0.02858	321	96507-97460	96806	0.06606	534	150122-150958	150540	0.01886
109	29762-30361	30061	0.028	322	96707-97718	97156	0.07352	535	150406-151173	150857	0.0157
110	29962-30561	30261	0.02609	323	96927-98031	97600	0.08519	536	150681-151373	151058	0.01107
111	30162-30764	30461	0.02453	324	97461-98407	97879	0.06461	537	150959-151573	151273	0.00382
112	30362-30964	30661	0.02122	325	97719-98608	98187	0.05813	538	151174-151776	151473	0.00424
113	30562-31236	30864	0.02866	326	98032-98814	98508	0.04449	539	151374-151976	151673	0.00531
114	30765-32041	31087	0.04784	327	98408-99038	98708	0.03618	540	151574-152176	151876	0.00362
115	30965-32404	31465	0.07615	328	98609-99256	98914	0.03687	541	151777-152377	152076	0.00321
116	31237-32719	32212	0.0833	329	98815-99465	99138	0.04188	542	151977-152585	152276	0.00361
117	32042-33519	32598	0.09465	330	99039-99727	99356	0.04135	543	152177-152785	152484	0.00515
118	32405-33818	33050	0.08515	331	99257-99927	99566	0.03485	544	152378-152993	152685	0.00432
119	32720-34907	33695	0.08685	332	99466-100220	99827	0.02387	545	152586-153193	152885	0.0031
120	33520-35255	33918	0.07711	333	99728-100420	100120	0.01638	546	152786-153393	153093	0.00298
121	33819-35544	35085	0.06642	334	99928-100637	100320	0.01066	547	152994-153593	153293	0.00218
122	34908-36697	35380	0.06712	335	100221-100867	100520	0.00801	548	153194-153793	153493	0.0028
123	35256-36942	35659	0.04842	336	100421-101078	100754	0.00768	549	153394-154047	153693	0.00395
124	35545-37152	36811	0.04666	337	100638-101283	100978	0.00639	550	153594-154275	153893	0.00697
125	36698-38152	37046	0.04935	338	100868-101483	101183	0.00639	551	153794-154528	154174	0.0109
126	36943-38655	38015	0.0611	339	101079-101741	101383	0.01117	552	154048-154756	154391	0.00951
127	37153-38991	38453	0.07979	340	101284-102055	101586	0.01658	553	154276-154972	154641	0.00776
128	38153-39606	38831	0.08229	341	101484-102287	101897	0.0174	554	154529-155185	154866	0.00333
129	38656-39904	39204	0.07401	342	101742-102631	102182	0.0276	555	154757-155429	155076	0.00353
130	38992-40104	39769	0.04764	343	102056-102883	102516	0.03111	556	154973-155788	155295	0.00596
131	39607-40304	40004	0.02807	344	102315-103086	102746	0.03556	557	155186-156014	155682	0.00682
132	39905-40504	40204	0.0194	345	102632-103286	102983	0.02592	558	155430-156239	155902	0.00772
133	40105-40704	40404	0.02233	346	102884-103507	103186	0.01965	559	155789-156541	156118	0.01466
134	40305-40904	40604	0.01847	347	103087-103743	103407	0.01745	560	156015-156741	156370	0.01464
135	40505-41104	40804	0.01616	348	103287-103955	103616	0.01238	561	156240-156941	156641	0.01302
136	40705-41304	41004	0.01674	349	103508-104158	103846	0.01172	562	156542-157141	156841	0.00253

137	40905-41504	41204	0.01942	350	103744-104361	104058	0.01392	563	156742-157341	157041	0.00135
138	41105-41704	41404	0.02379	351	103956-104561	104258	0.01462	564	156942-157541	157241	0.00133
139	41305-41904	41604	0.02347	352	104159-104803	104461	0.01649	565	157142-157741	157441	0.00117
140	41505-42104	41804	0.02214	353	104362-105015	104664	0.01712	566	157342-157946	157641	0.00133
141	41705-42304	42004	0.01987	354	104562-105371	104903	0.01733	567	157542-158183	157846	0.00205
142	41905-42630	42204	0.0337	355	104804-105577	105130	0.01803	568	157742-158468	158050	0.00606
143	42105-43032	42413	0.05751	356	105025-105795	105474	0.01251	569	157947-158761	158312	0.01481
144	42305-43295	42738	0.06592	357	105372-105998	105695	0.01501	570	158184-159044	158603	0.0279
145	42631-43513	43195	0.0645	358	105578-106219	105898	0.01392	571	158469-159364	158911	0.0308
146	43033-43862	43395	0.05584	359	105796-106458	106101	0.01618	572	158762-159640	159247	0.02894
147	43296-44235	43711	0.0586	360	105999-106673	106349	0.01799	573	159045-160132	159503	0.02168
148	43544-44486	44128	0.05136	361	106220-106885	106570	0.01657	574	159365-160338	160011	0.01611
149	44000-44716	44386	0.04435	362	106459-107132	106779	0.01741	575	159641-160538	160238	0.00941
150	44236-44931	44592	0.03838	363	106674-107335	107002	0.01365	576	160133-160766	160438	0.00546
151	44487-45131	44831	0.02869	364	106886-107535	107232	0.01298	577	160339-160972	160658	0.00612
152	44721-45331	45031	0.0208	365	107133-107738	107435	0.00848	578	160539-161178	160872	0.00895
153	44932-45531	45231	0.02104	366	107336-108136	107638	0.01179	579	160767-161378	161078	0.00943
154	45132-45731	45431	0.0251	367	107536-108345	108027	0.01575	580	160973-161590	161278	0.01103
155	45332-45931	45631	0.02762	368	107739-108545	108236	0.01581	581	161179-161848	161478	0.01039
156	45532-46131	45831	0.0261	369	108137-108751	108445	0.01279	582	161379-162048	161702	0.00885
157	45732-46331	46031	0.02602	370	108346-108969	108645	0.01002	583	161591-162248	161948	0.00639
158	45932-46531	46231	0.0299	371	108546-109193	108863	0.01057	584	161849-162448	162148	0.00419
159	46132-46731	46431	0.02498	372	108752-109654	109078	0.01236	585	162049-162655	162348	0.00454
160	46332-46931	46631	0.02134	373	108970-109863	109296	0.01526	586	162249-162874	162548	0.00483
161	46532-47131	46831	0.01018	374	109194-110081	109763	0.01501	587	162449-163094	162755	0.00754
162	46732-47331	47031	0.00827	375	109655-110938	109975	0.01776	588	162656-163298	162980	0.00776
163	46932-47531	47231	0.00797	376	109864-111446	110822	0.01693	589	162875-163498	163198	0.00735
164	47132-47737	47431	0.01657	377	110082-111671	111065	0.02017	590	163095-163698	163398	0.00791
165	47332-47937	47631	0.01969	378	110939-111874	111571	0.01307	591	163299-163898	163598	0.00694

166	47532-48137	47837	0.02123	379	111447-112074	111774	0.01051	592	163499-164126	163798	0.00846
167	47738-48337	48037	0.01632	380	111672-112274	111974	0.00626	593	163699-164631	164001	0.01243
168	47938-48537	48237	0.01657	381	111875-112478	112174	0.00684	594	163899-164966	164451	0.01816
169	48138-48737	48437	0.016	382	112075-112692	112374	0.00645	595	164127-165709	164738	0.02034
170	48338-48937	48637	0.01848	383	112275-112917	112582	0.00879	596	164632-165918	165600	0.01828
171	48538-49137	48837	0.01803	384	112479-113124	112808	0.00984	597	164967-166382	165812	0.01739
172	48738-49359	49037	0.02096	385	112693-113324	113024	0.00702	598	165710-166606	166024	0.01435
173	48938-49623	49237	0.02652	386	112918-113524	113224	0.00472	599	165919-166824	166488	0.01228
174	49138-50223	49495	0.0493	387	113125-113724	113424	0.00384	600	166383-167030	166709	0.0099
175	49360-50428	49964	0.05176	388	113325-113977	113624	0.00606	601	166607-167230	166930	0.00973
176	49624-50706	50323	0.05908	389	113525-114194	113824	0.00801	602	166825-167439	167130	0.01236
177	50224-51000	50573	0.04154	390	113725-114394	114094	0.00947	603	167031-167717	167330	0.01135
178	50429-51299	50823	0.03961	391	113978-114594	114294	0.00957	604	167231-168040	167539	0.01587
179	50707-51506	51122	0.02404	392	114195-114804	114494	0.00908	605	167440-168240	167937	0.01253
180	51001-51762	51399	0.02581	393	114395-115029	114703	0.01072	606	167718-168443	168140	0.01327
181	51300-51997	51643	0.03252	394	114595-115234	114917	0.00895	607	168041-168673	168340	0.0124
182	51507-52287	51866	0.04644	395	114805-115434	115134	0.00786	608	168241-168900	168564	0.01394
183	51763-52524	52127	0.04437	396	115030-115915	115334	0.00805	609	168444-169115	168788	0.01756
184	51998-53814	52405	0.03604	397	115235-116202	115552	0.01508	610	168674-169354	169003	0.01653
185	52288-54244	53681	0.03745	398	115435-116494	116051	0.02135	611	168901-169575	169215	0.01844
186	52525-54444	54138	0.04036	399	115916-116766	116308	0.02708	612	169116-169775	169460	0.01427
187	53824-54644	54344	0.03771	400	116203-117054	116643	0.03019	613	169355-169978	169675	0.0152
188	54245-54931	54544	0.03287	401	116495-117355	116911	0.031	614	169576-170199	169878	0.01419
189	54445-55636	54747	0.04398	402	116767-117610	117206	0.02184	615	169776-170546	170096	0.01314
190	54645-56263	55262	0.06364	403	117055-117815	117473	0.011	616	169979-170770	170302	0.01606
191	54932-56490	55978	0.05574	404	117356-118015	117710	0.00257	617	170200-171009	170661	0.01754
192	55637-56818	56379	0.05002	405	117611-118215	117915	0.00068	618	170547-171212	170873	0.01862
193	56264-57182	56718	0.05144	406	117816-118415	118115	0.00117	619	170771-171415	171112	0.01784
194	56491-57648	56923	0.0548	407	118016-118615	118315	0.00133	620	171010-171618	171312	0.01478

195	56819-58408	57501	0.061	408	118216-118815	118515	0.00199	621	171213-171827	171515	0.01302
196	57183-58616	57764	0.04274	409	118416-119015	118715	0.00185	622	171416-172057	171724	0.00988
197	57649-58816	58516	0.04568	410	118616-119305	118915	0.00559	623	171619-172269	171936	0.01211
198	58409-59075	58716	0.0369	411	118816-119551	119115	0.0078	624	171828-172487	172166	0.01737
199	58617-59309	58917	0.04163	412	119016-119767	119451	0.00947	625	172058-172690	172387	0.02004
200	58817-59509	59209	0.04057	413	119320-119987	119663	0.00784	626	172270-172939	172590	0.0218
201	59079-59709	59409	0.03248	414	119552-120378	119881	0.00694	627	172488-173266	172793	0.03043
202	59310-59931	59609	0.03242	415	119768-120597	120250	0.00614	628	172691-173532	173054	0.02976
203	59510-60131	59831	0.03243	416	119988-120804	120486	0.00515	629	172940-173846	173418	0.02888
204	59710-61456	60031	0.04841	417	120379-121028	120697	0.00419	630	173267-174115	173686	0.01719
205	59932-61684	60250	0.04956	418	120598-121281	120920	0.00793	631	173533-174315	173995	0.01519
206	60132-61955	61579	0.05375	419	120805-121504	121163	0.00828	632	173847-174515	174215	0.00977
207	61457-62270	61825	0.04819	420	121029-121771	121393	0.0106	633	174116-174731	174415	0.00766
208	61697-62529	62104	0.05234	421	121282-121971	121671	0.00633	634	174316-174947	174620	0.00546
209	61956-62974	62423	0.05678	422	121505-122171	121871	0.00471	635	174516-175178	174844	0.00821
210	62271-63227	62736	0.04769	423	121772-122371	122071	0.0028	636	174732-175378	175074	0.00801
211	62530-63451	63116	0.03788	424	121972-122571	122271	0.00218	637	174948-175667	175278	0.0109
212	62975-63664	63338	0.02523	425	122172-122779	122471	0.00298	638	175179-177251	175381	0.01092
213	63228-63864	63551	0.02272	426	122372-122979	122679	0.0031				
