# Supplementary material for: Complete chloroplast genome sequence and phylogenetic analysis of Symphytum officinale
Source: Genet Mol Biol. 2025 Jun 30;48(2):e20240258. doi: 10.1590/1678-4685-GMB-2024-0258 (PMC12210358; doi:10.1590/1678-4685-GMB-2024-0258)
Supplement: Figure S1 - [file 1415-4757-GMB-48-2-e20240258-s7.pdf]

Genetics and Molecular Biology
https://doi.org/10.1590/1678-4685-GMB-2024-0258

Supplementary Material to: Complete chloroplast genome sequence and phylogenetic analysis of *Symphytum officinale*

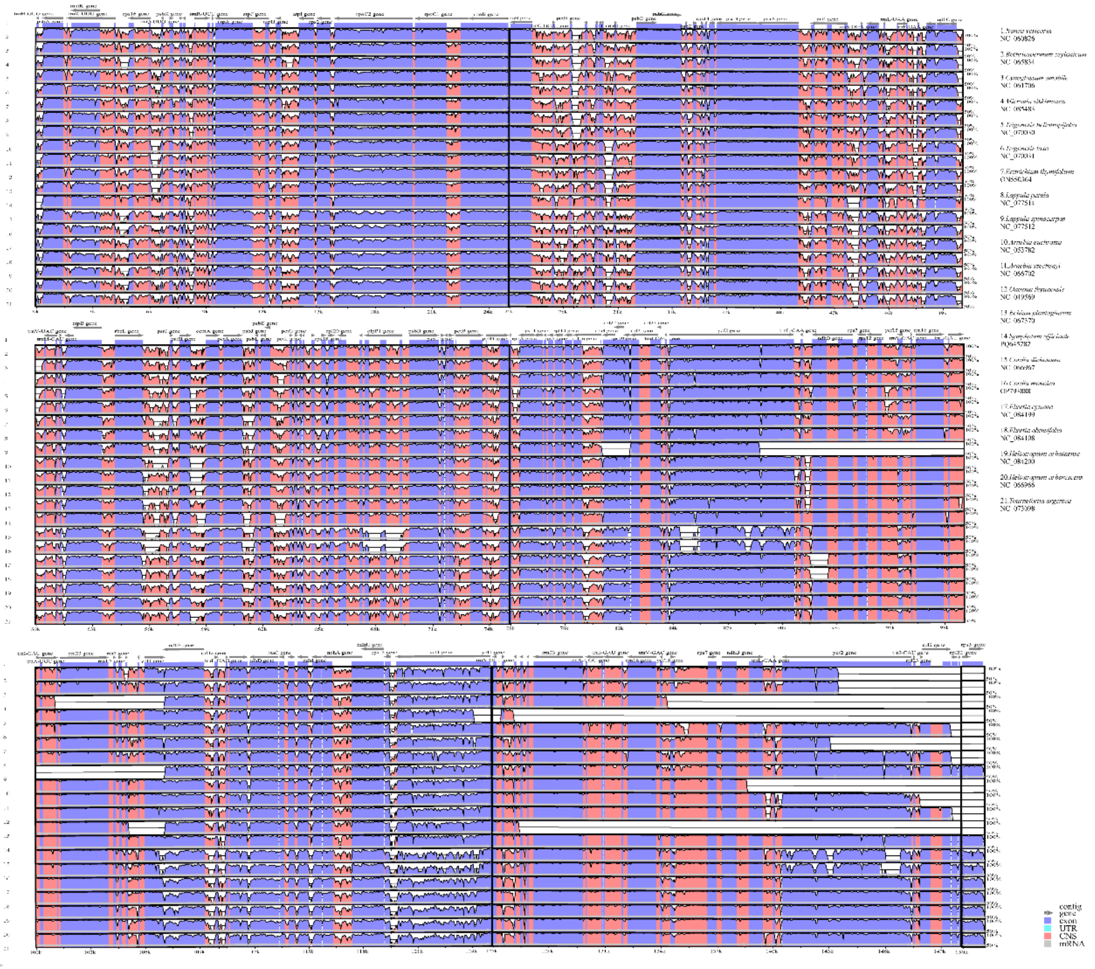

Figure S1- Sequence identity plot comparing the 21 Boraginaceae chloroplast genomes with Nonea vesicaria as a reference by using mVISTA. The y-axis represents % identity ranging from 50 to 100%. Coding and non-coding regions are marked in purple and pink, respectively.
