# Supplementary material for: Complete chloroplast genome sequence and phylogenetic analysis of Symphytum officinale
Source: Genet Mol Biol. 2025 Jun 30;48(2):e20240258. doi: 10.1590/1678-4685-GMB-2024-0258 (PMC12210358; doi:10.1590/1678-4685-GMB-2024-0258)
Supplement: Figure S2 - [file 1415-4757-GMB-48-2-e20240258-s8.pdf]

Genetics and Molecular Biology
https://doi.org/10.1590/1678-4685-GMB-2024-0258

Supplementary Material to: Complete chloroplast genome sequence and phylogenetic analysis of *Symphytum officinale*

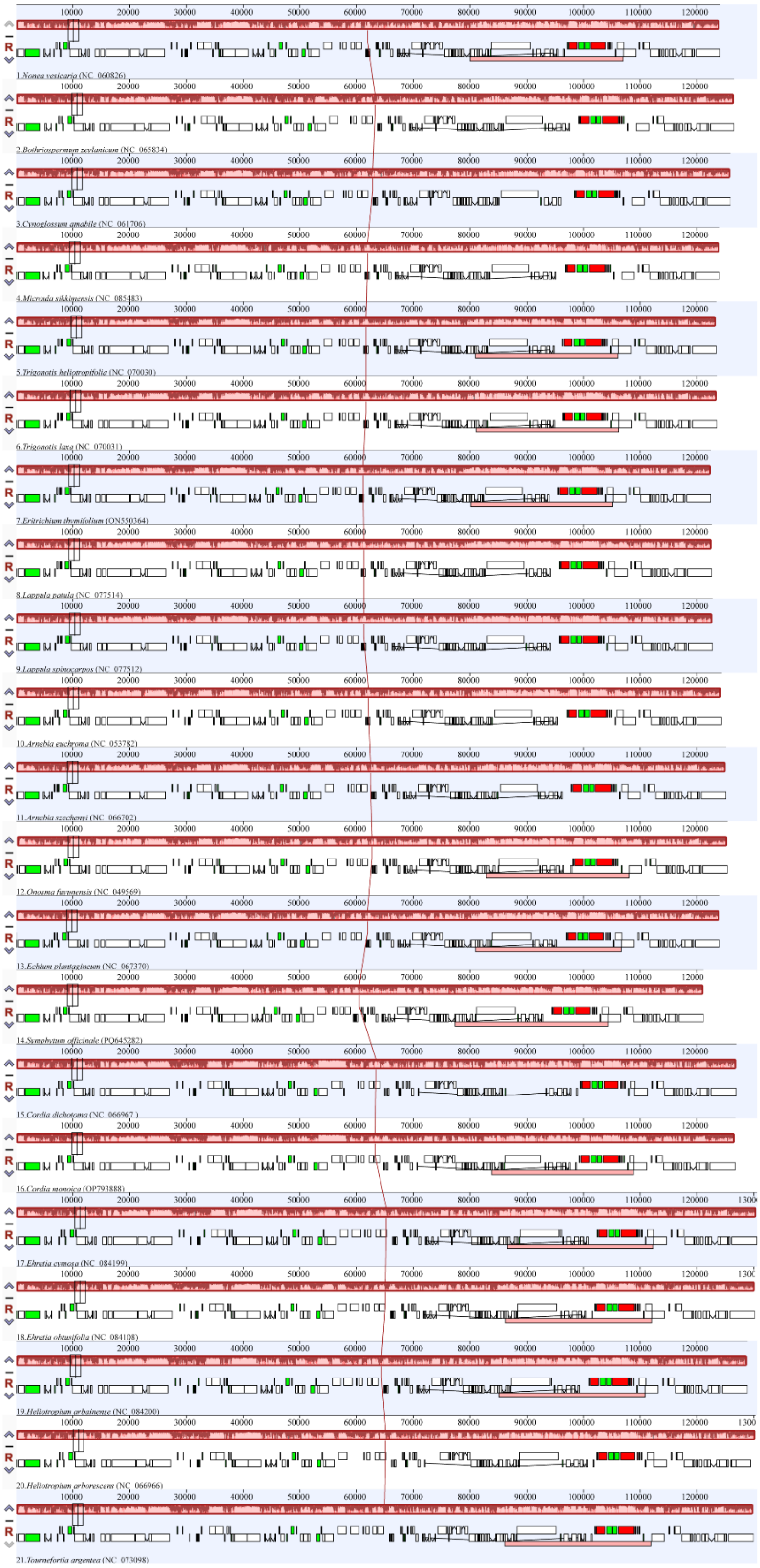

Figure S2 - MAUVE alignment of 21 Boraginaceae species chloroplast genomes, The Nonea vesicaria genome is shown at top as the reference genome. Within each of the alignments, local collinear blocks are represented by blocks of the same color connected by lines.
